# Targeting Pro-Survival Autophagy Enhanced GSK-3β Inhibition-Induced Apoptosis and Retarded Proliferation in Bladder Cancer Cells

**DOI:** 10.3390/curroncol30060406

**Published:** 2023-05-28

**Authors:** Yuko Shirono, Vladimir Bilim, Tsutomu Anraku, Hiroo Kuroki, Akira Kazama, Masaki Murata, Kaede Hiruma, Yoshihiko Tomita

**Affiliations:** 1 Department of Urology, Division of Molecular Oncology, Graduate School of Medical and Dental Sciences, Niigata University, Niigata 951-8510, Japan; vbilim@zoho.com (V.B.); exfeel@live.jp (A.K.);; 2 Department of Urology, Kameda Daiichi Hospital, Niigata 950-0165, Japan; 3 Department of Urology, Sado General Hospital, Sado 952-1209, Japan; 4 Glickman Urological and Kidney Institute, Cleveland Clinic, Cleveland, OH 44195, USA

**Keywords:** AMPK, autophagy, bladder cancer, GSK-3β, LC3B, TFEB, ULK1

## Abstract

Advanced bladder cancer (BC) (local invasive and/or metastatic) is not curable even with cytotoxic chemotherapy, immune checkpoint inhibitors, and targeted treatment. Targeting GSK-3β is a promising novel approach in advanced BC. The induction of autophagy is a mechanism of secondary resistance to various anticancer treatments. Our objectives are to investigate the synergistic effects of GSK-3β in combination with autophagy inhibitors to evade GSK-3β drug resistance. Small molecule GSK-3β inhibitors and GSK-3β knockdown using siRNA promote the expression of autophagy-related proteins. We further investigated that GSK-3β inhibition induced the nucleus translocation of transcription factor EB (TFEB). Compared to the GSK-3β inhibition alone, its combination with chloroquine (an autophagy inhibitor) significantly reduced BC cell growth. These results suggest that targeting autophagy potentiates GSK-3β inhibition-induced apoptosis and retarded proliferation in BC cells.

## 1. Introduction

Bladder cancer (BC) is the 10th most frequent cancer globally, accounting for 573,278 new cases and 212,536 deaths in 2020 [1]. Urothelial cancer (UC) is the most common type of BC, accounting for approximately 90% of all cases.

Although non-muscle invasive bladder cancer (NMIBC) reflects a large proportion of all BCs and is commonly associated with a good prognosis, locally advanced or metastatic BCs are mainly associated with death. Although there was no treatment beyond cisplatin-based chemotherapy for a long time, pembrolizumab was introduced for advanced UC that recurred or progressed after platinum-based chemotherapy. The objective response rate in the international phase III KEYNOTE-045 clinical trials for pembrolizumab was around 21.1%, as compared with 11.4% in the chemotherapy group [2]. Despite currently available multimodality therapy, advanced BC has a very low overall survival (OS) rate of about 6% [3].

The drug pricing is high relative to the response rates, and the development of new therapeutic agents is required to improve the clinical outcomes of patients with locally advanced or metastatic BC.

Glycogen synthase kinase-3β (GSK-3β) is a serine/threonine protein kinase that was first described as a component of glycogen synthase (GS) regulation through its phosphorylation [4]. GSK-3 has two isoforms, GSK-3α and GSK-3β [5,6], which exhibit 97% sequence identity within their catalytic domains [7,8]. We previously identified GSK-3 as a potential therapeutic target for BC and reported that the antitumor effects of GSK-3β inhibitors are attributed to enhanced apoptosis [9,10]. GSK-3β inhibitors also promote autophagy, and the inhibition of autophagy by chloroquine enhances the antitumor effects of GSK-3β inhibitors [10,11,12]. GSK-3β inhibitors were proven to induce autophagy in other cancer types. These previous studies led us to investigate the induction of autophagy as not yet clarified by GSK-3β inhibitors in BC cells, as well as the combined effects of autophagy inhibitors and GSK-3β inhibitors. Currently, phase I and II trials using Elraglusib (a novel highly specific GSK-3 inhibitor) are underway [13].

Autophagy is an evolutionarily ancient mechanism that culminates with the lysosomal degradation of superfluous or potentially dangerous cytosolic entities [14]. During the initial stage of cancer development, autophagy serves a major role in tumor suppression by maintaining genomic integrity and preventing proliferation and inflammation [15]. However, after the establishment of cancer, cancer cells may utilize autophagy to survive cellular stresses in the adverse microenvironment [16].

The exact mechanism of autophagy induction by GSK-3 inhibition in UC cells is not clear. One of the autophagy signaling pathways is the AMP-AMPK-ULK1 pathway. AMP-activated protein kinase (AMPK) phosphorylates Unc-51-like autophagy, activating kinase 1 (ULK1), an important initiator of autophagy. Furthermore, AMPK is known to inhibit the mammalian target of rapamycin complex 1 (mTORC1), and mTORC1 inhibits the activation of ULK1 and regulates the transcription of autophagy-related genes [17].

We next focused on transcription factor EB (TFEB). TFEB regulates various cellular processes, including autophagy [18], lysosomal biogenesis [19], and energy metabolism [20]. It is known that mTORC1 inhibits TFEB nuclear translocation [21]. Under normal nutritionally replete conditions, mTORC1 that is present on the lysosomal membrane phosphorylates TFEB, and this phosphorylation inhibits the translocation of TFEB to the nucleus and prevents its function as a transcription factor [21]. Under cellular stress, such as low levels of ATP or glucose, mTORC1 is inhibited, and TFEB then translocates to the nucleus to transcribe its target genes [21]. Once in the nucleus, TFEB is responsible for the transcription of autophagy-related genes and genes encoding lysosomal proteins [22]. Once transcribed and translated, the autophagy-related proteins are responsible for the formation of the double membrane autophagosome, in which damaged proteins and organelles are sequestered [23,24]. The autophagosome loaded with cargo fuses with the lysosome, resulting in the formation of the autolysosome [25]. The acidic hydrolases from lysosomes degrade the loaded cargo and release nutrients back into the cytoplasm for recycling, correcting the nutritional deficiency [25].

Chloroquine, an anti-malarial drug, is the most frequently used agent for the inhibition of autophagy. Chloroquine and its derivative hydroxychloroquine are the only clinically available autophagy inhibitors approved by the US Food and Drug Administration [26]. Several published studies indicate that chloroquine can sensitize cancer cells to chemotherapy and radiotherapy, that is to say, enhancing the anticancer effect [11,12,27]. These findings suggest that autophagy is induced by chemotherapy and radiotherapy as a protective mechanism in BC cells, and that the inhibition of autophagy can enhance therapeutic sensitivity.

## 2. Materials and Methods

### 2.1. Cells and Cell Culture

Bladder cancer cell lines T24, HT1376, RT4, and human embryonic kidney 293 (HEK293) cells were purchased from the American Type Culture Collection (ATCC, Manassas, VA, USA). The cells were cultured in RPMI-1640 medium (Gibco; Thermo Fisher Scientific, Inc., Grand Island, NY, USA) supplemented with 10% FBS (Gibco; Thermo Fisher Scientific, Inc.), 1% MEM non-essential amino acids (Gibco; Thermo Fisher Scientific, Inc.), 1% MEM sodium pyruvate solution 100 mM (Gibco; Thermo Fisher Scientific, Inc.), and 90 µg/mL kanamycin in a 37 °C incubator containing 5% CO_2_.

Passaging was achieved using 5 mL 0.05% Trypsin-EDTA (Gibco; Thermo Fisher Scientific, Inc.) when cells were fused to 80%. AR-A014418 and TDZD-8 (GSK-3 inhibitor) were purchased from Abcam(Cambridge, UK).

### 2.2. Cell Viability Assay

The cells were seeded in 96-well plates at 1–2 × 10^3–4^ cells/well with 100 µL medium for 24 h, and then treated with AR-A014418 at the indicated concentrations (0, 0.5, 1, 5, 10, and 25 µM) for 24, 48, and 72 h. Following incubation, 10 µL CellTiter 96^®^ Aqueous One Solution Regent (Promega Corporation, Madison, WI, USA) was added into each well and incubated for 2 h. The absorbance was measured at 490 nm using the iMark^TM^ 96-well microplate reader (Bio-Rad Laboratories, Inc., Hercules, CA, USA). Chloroquine (phosphate) (14194) was purchased from the Cayman chemical company (Ann Arbor, MI, USA).

### 2.3. Protein Extraction and Western Blot Analysis

Subconfluent cell cultures were washed with cold PBS and lysed in lysis buffer (20 mM Tris-HCl (pH 7.5), 150 mM NaCl, 1 mM Na_2_EDTA, 1 mM EGTA, 1% Triton, 2.5 mM sodium pyrophosphate, 1 mM beta-glycerophosphate, 1 mM Na_3_VO_4_, 1 µg/mL).

Following clarification of the lysates by centrifugation at 15,000× *g* for 30 min at 4 °C, protein concentration was detected using the Bradford method, and 20–30 µg of each protein was electrophoretically separated on a 4–15% or 10 or 7.5% SDS-polyacrylamide gel and transblotted to a PVDF membrane. Immunoblots were blocked with 10% skimmed milk in TBS, followed by incubation with primary antibodies. Horseradish peroxidase-labeled ECL™ Anti-mouse IgG (1:5000) and ECL™ Anti-rabbit IgG (1:10,000) from GE Healthcare(Piscataway, NJ, USA) were used as secondary antibodies and detected using Clarity Max Western ECL Substrate from Bio-Rad Laboratories, Inc. according to the manufacturer’s instructions. The expression of β-actin (Cell Signaling Technology, Inc., Danvers, MA, USA, 12262) was used as a loading control. The images were analyzed using Ez-Capture MG (Atto Corporation, Tokyo, Japan). The following primary antibodies were purchased from Cell Signaling Technology, Inc.: SQSTM1/p62 (8025), Beclin-1 (3738), p-Beclin-1 (S15) (84966), p-Beclin-1 (S93) (14717), LC3 (3868), GSK3β (12456), TFEB (4240), α-Tubulin (9099), Histone3 (12648), p-ULK1 (14202), ULK1 (8054), p-AMPKα (2535), AMPKα (5831).

The dilution ratios of the primary antibodies were 1:200–1:2000.

According to the manufacturer’s instructions, nuclear/cytosolic fractionation was carried out using the MinuteTM Cytoplasmic and Nuclear Extraction Kit for Cells (Invent Biotechnologies, Inc., Plymouth, MN, USA, SC-003). α-Tubulin and Histone3 were used as a control for cytosolic and nuclear lysates, respectively.

### 2.4. Estimation of Autophagic Flux

pMRX-IP-GFP-LC3-RFP-LC3ΔG (plasmid # 84572) was purchased from Addgene, Inc. (Watertown, MA, USA). The plasmid was transformed into NEB^®^ stable Competent *E. coli* (New England Biolabs, Inc., Ipswich, MA, USA, C3040H), and plasmid DNA was extracted and purified using PureLink^TM^ HiPure Plasmid Midiprep Kit (Invitrogen; Thermo Fisher Scientific, Inc., Carlsbad, CA, USA).

The cells (70–90% confluent) were grown in 24-well plates and transfected with the plasmid according to the Lipofectamine^TM^ 3000 Reagent protocols. After 24 h of incubation, the cells were treated with 10 µM AR-A014418 for 12, 24, and 48 h, and images were captured with fluorescence microscope (Olympus, Tokyo, Japan, IX71) at 40× magnification. The absorption filters used were BA510IF for green fluorescence and BA575IF for red fluorescence. The fluorescence intensity was quantified using the ImageJ imaging software program (version 1.53t). 

### 2.5. Nuclear Translocation of TFEB-EGFP

pEGFP-N1-TFEB (plasmid#38119) was purchased from Addgene, Inc. The plasmid was transformed into competent high DH5α (TOYOBO Co., Ltd., Osaka, Japan, DNA-903), and plasmid DNA was extracted and purified using PureLink^TM^ HiPure Plasmid Midiprep Kit (Invitrogen; Thermo Fisher Scientific, Inc.).

The cells (70–90% confluent) were grown in 24-well plates and transfected with the plasmid according to the Lipofectamine^TM^ 3000 Reagent protocols. After 24 h of incubation, the cells were treated with 10 µM AR-A014418 for 24 h, and nuclear translocation of TFEB-EGFP was examined and analyzed via fluorescent microscopy (Olympus, IX71) at 40× magnification. The absorption filters used were BA510IF for green fluorescence. The nuclei were stained with Hoechst 33342 (Bio-Rad Laboratories, Inc., 1351304) 5 µg/mL for 10 min at room temperature.

### 2.6. siRNA Transfection

For GSK-3β silencing, the cells were transfected with specific human siRNAs against GSK-3β by using Lipofectamine RNAiMAX (Invitrogen, Thermo Fisher Scientific Inc.) according to the manufacturer’s recommendations. siRNA targeting human GSK-3β (s6239, s6241) and control siRNA with a scrambled sequence (AM4611) were purchased from Ambion, Thermo Fisher Scientific, Inc. (Austin, TX, USA). Targeting sequence of siRNA are as follows: GSK-3β (s6239); CUCAAGAACUGUCAAGUAAtt, GSK-3β (s6241); GCUAGAUCACUGUAACAUAtt. After 48 h post-transfection, proteins were extracted. The knockdown efficiency was then measured using Western blot analysis.

### 2.7. Statistical Analysis

Continuous variables are presented as the mean ± SD. All continuous variables in this study met the criteria for normal distribution and were assumed to be parametric. Data were analyzed using one-way ANOVA with Dunnett’s test for multiple comparisons. Statistical analysis was performed using GraphPad Prism software (version 8.0) (GraphPad Software, Inc., San Diego, CA, USA). *p* < 0.05 was considered to indicate a statistically significant difference.

## 3. Results

### 3.1. GSK-3 Inhibits the Proliferation of Bladder Cancer Cells and Has Low Cytotoxicity in Normal Cells

**Figure 1:**
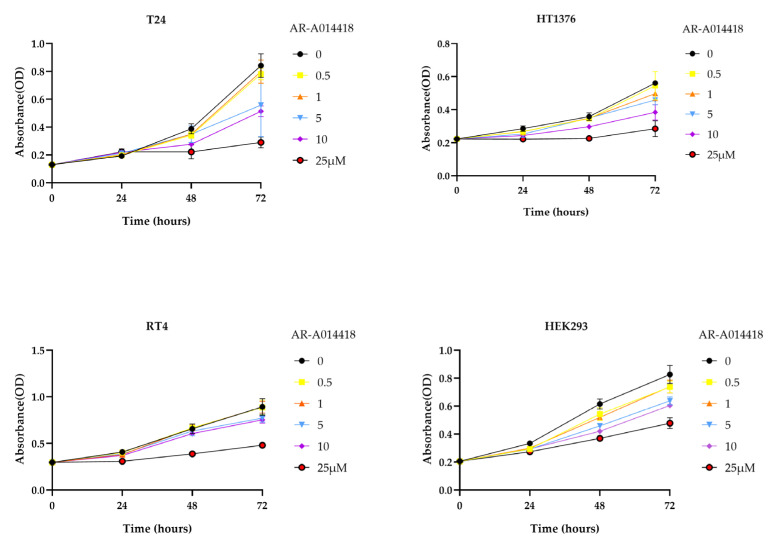
AR-A014418 suppressed the viability of bladder cancer cells. Relative cell viability was measured using MTS assay in T24, HT1376, RT4, and human embryonic kidney 293 (HEK293) cells treated with the indicated concentration of AR-A014418 for 24, 48, and 72 h. OD; optical density. Data are shown as mean ± SD of four independent experiments.

To study the effect of AR-A014418 (GSK-3β inhibitor) on the growth of cells in vitro, we used T24 and HT1376, high grade UC cell lines, RT4, a low grade UC cell line, and human embryonic kidney 293 (HEK293) cells. The MTS assay was used to observe the effects of AR-A014418 on cell proliferation. Our results indicate that AR-A014418 inhibits the growth of T24, HT1376, and RT4 cells after 24–72 h of treatment (Figure 1), and that the growth of BC cells is inhibited in a dose- and time-dependent manner. Moreover, AR-A014418 does not cause toxicity to normal kidney cells comparatively. Similarly, cell proliferation was examined with another GSK-3β inhibitor, TDZD-8, using MTS assay. It showed no significant difference compared to the control up to 48 h after TDZD-8 treatment, but showed significant differences at 72 h after treatment, as shown in Supplementary Figure S1.

### 3.2. GSK-3 Inhibition Induces Autophagy in Cultured Bladder Cancer Cells

**Figure 2:**
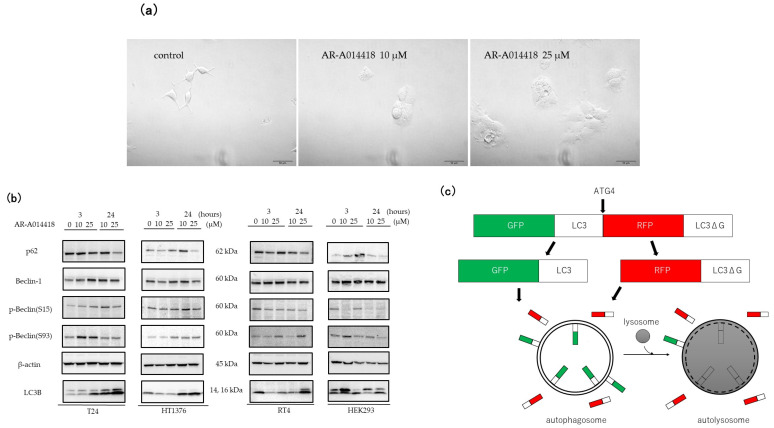

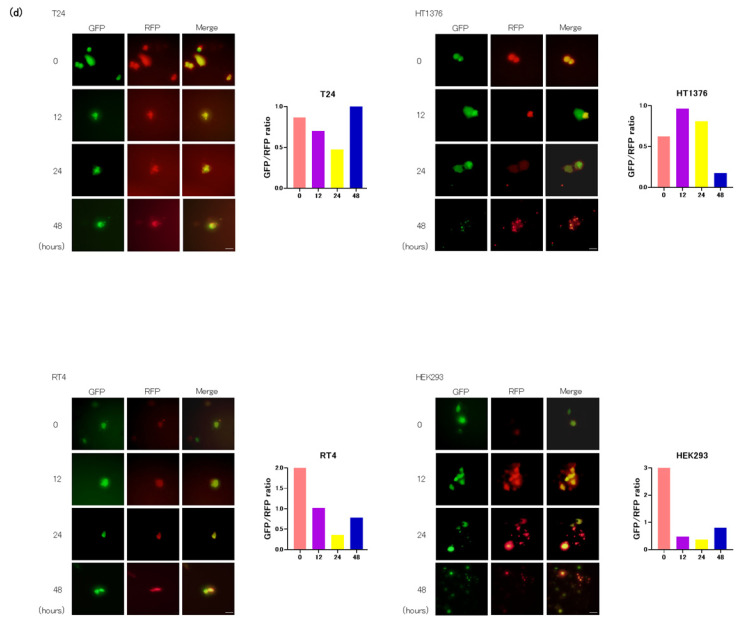
Effect of AR-A014418 on the expression of autophagy-related proteins in bladder cancer cells. (**a**) Brightfield images of HT1376 treated with 0 (control), 10, and 25 µM AR-A014418 for 24 h. Scale bar = 50 µm. (**b**) Western blot analysis of p62, Beclin-1, p-Beclin (s15, s93), and LC3B. β-actin was used as a loading control. The original blots are presented in Supplementary Figure S4a–d. (**c**) Schematic illustration of the measurement of autophagic flux with a GFP-LC3-RFP-LC3ΔG probe. (**d**) All four cell lines expressing GFP-LC3-RFP-LC3ΔG were treated with 10 µM AR-A014418 for designated times. Images were taken with a fluorescence microscope. GFP/RFP ratio data were quantified using ImageJ and are shown next to the images. Scale bar = 50 µm.

The cells were treated with 10 and 25 μM AR-A014418 for 24 h, and the morphological changes were investigated under an inverted microscope. As shown in Figure 2a, compared with the control group, AR-A014418-treated cells showed an increased accumulation of cytoplasmic vacuoles and the formation of autophagosome-like structures in the cytoplasm.

To determine whether AR-A014418 induces autophagy in BC cells, we examined the expression of autophagy-related proteins, including p62, Beclin-1, p-Beclin (S15), p-Beclin (S93), and LC3B (Figure 2b). LC3B is the most widely used autophagosome marker because the amount of LC3B-II reflects the number of autophagosomes and autophagy-related structures. In addition, the degradation of p62 is another widely used marker to monitor autophagic activity because p62 directly binds LC3B and is selectively degraded by autophagy [28,29], and Beclin-1 is the main autophagy gene associated with cancer, and it was reported that autophagy might inhibit or promote tumorigenesis by focusing on Beclin-1 [30,31]. After treatment with the indicated concentrations of AR-A014418 for 0, 3, and 24 h, the Western blot analysis showed a marked increase in LC3B in a dose- and time-dependent manner except for the normal kidney cells. Consistent with an increase in autophagy, p62 showed the reverse response to high concentrations (25 μM) of AR-A014418. The levels of Beclin-1 were higher at 24 h than at 3 h at low concentrations (10 μM), while the expression remained unchanged at high concentrations in T24 and HT1376, and no significant changes were observed in RT4 and HEK293 compared to the controls. No consistent trend was found in p-Beclin.

Similarly, a Western blot analysis was performed with TDZD-8, as shown in Supplementary Figure S2. The level of LC3B increased in a dose- and time-dependent manner except for the normal kidney cells, but no consistent trend was found in p62 and Beclin-1.

We used a GFP-LC3-RFP-LC3ΔG probe to observe the stepwise progression of autophagy. When GFP-LC3-RFP-LC3ΔG is expressed in cells, ATG4 cleaves the C-terminus of the wild type LC3, forming GFP-LC3-I, and releasing RFP-LC3-ΔG to the cytosol. While RFP-LC3-ΔG remains intact in the cytosol, GFP-LC3-I can be conjugated to PE on autophagic vesicle membranes, and a fraction of it is degraded in autolysosomes [32] (Figure 2c). In short, autophagic activity can be simply and quantitatively estimated by determining the ratio of the GFP and RFP signal intensities. All four cell lines stably expressing GFP-LC3-RFP-LC3ΔG were treated with 10 μM AR-A014418 for 0, 12, 24, and 48 h. The GFP/RFP ratio was lower in all the cells treated for 24 h compared to those treated for 12 h. The decline in the GFP/RFP ratio indicates autophagic flux. In HT1376 and HEK293, the cells shrunk, and apoptotic bodies formed after 48 h of treatment (Figure 2d).

**Figure 3:**
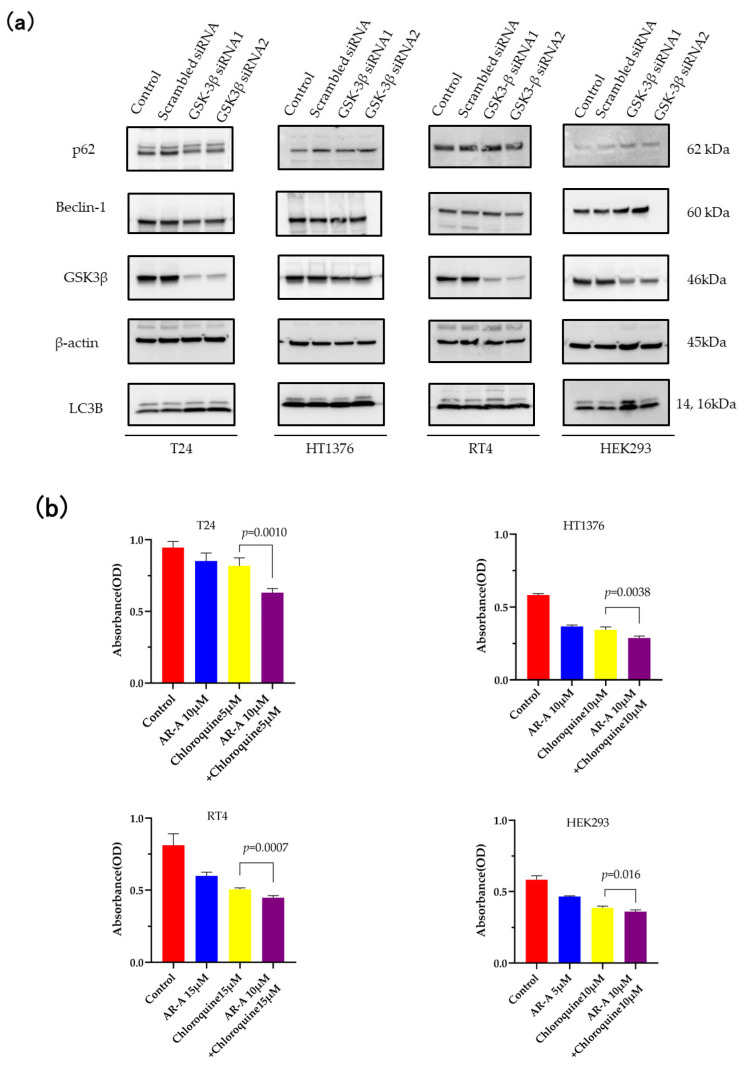
GSK-3β knockdown efficiency of siRNA. Synergistic inhibitory effect of autophagy inhibitor and AR-A014418. (**a**) Western blot analysis of GSK-3β, autophagy-related proteins, and β-actin as a loading control. The original blots are presented in Supplementary Figure S4e–h. Effect of a synergistic inhibitory effect of autophagy inhibitor and AR-A014418. (**b**) Relative cell viability was measured using MTS assay in bladder cancer cell lines treated with AR-A014418 (AR-A) in combination with autophagy inhibitor chloroquine for 24 h as indicated. Columns, mean; bars, SD. *p* values are indicated above the pairwise brackets. Statistical analysis was performed using one-way ANOVA with Dunnett’s test.

To further confirm the mechanism of GSK-3β inhibition to induce autophagy, we used the GSK-3β knockdown using siRNA in BC cells. In these experiments, a scrambled siRNA sequence was used as a negative control, and lipofectamine was only added as a control. The knockdown efficiency of siRNA in all four cell lines was proven 48 h post-siRNA transfection via Western blot analysis (Figure 3a). It should be noted that the efficiency of GSK-3β knockdown was particularly weak in HT1376 compared to the other BC cells, as shown by the densitometry readings/intensity ratio of GSK-3β in Supplementary Figure S4r. GSK-3β knockdown promoted the expression of LC3B and the degradation of p62 in T24, and promoted the expression of LC3B by one of the siRNAs in HEK293. However, the expression of Beclin-1 remained unchanged in all four cells.

### 3.3. Inhibition of Autophagy Sensitizes the Cells to GSK-3 Inhibition-Induced Apoptosis

We showed in the previous sections that AR-A014418 suppresses cell proliferation and induces autophagy. Further, we explored whether this autophagy induction is cytoprotective or cytotoxic for BC cells by inhibiting autophagy with chloroquine.

The MTS assay revealed that the combination of AR-A014418 and chloroquine significantly suppressed the viability of T24 (*p* = 0.001), HT1376 (*p* = 0.0038), RT4 (*p* = 0.0007), and HEK293 (*p* = 0.016), compared with the BC cells treated with AR-A014418 or chloroquine alone (Figure 3b). Next, we observed the morphological changes in the GSK-3β knockdown using siRNA. The siGSK-3β-transfected BC cells treated with chloroquine for 24 h showed extensive vacuolation, and for 48 h, they tended to show nuclear degeneration. However, no such tendency toward cell vacuolation or nuclear degeneration was observed in HEK293 (Supplementary Figure S3). It was shown that AR-A014418 induces autophagy in BC cells, and its inhibition suppresses cell proliferation. In other words, this form of autophagy is cytoprotective and reduces cell death.

### 3.4. TFEB Nuclear Translocation Is Governed by GSK-3 Inhibition

Apart from autophagy initiation mediated by four signal-sensing kinases, mTORC1, ULK1, AMPK, and protein kinase B (AKT), autophagy is also regulated by transcriptional and epigenetic mechanisms. The transcription factor EB (TFEB), a master gene for lysosomal biogenesis, coordinates this program by driving the expression of autophagy and lysosomal genes. TFEB modulates the formation of autophagosomes and the fusion of the autophagosome and lysosome [33].

**Figure 4:**
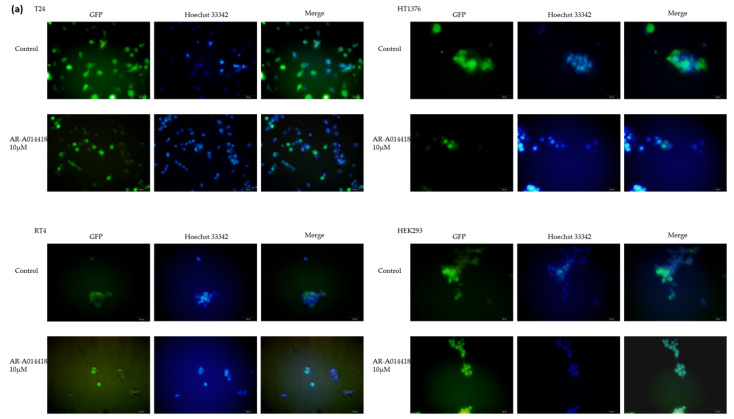

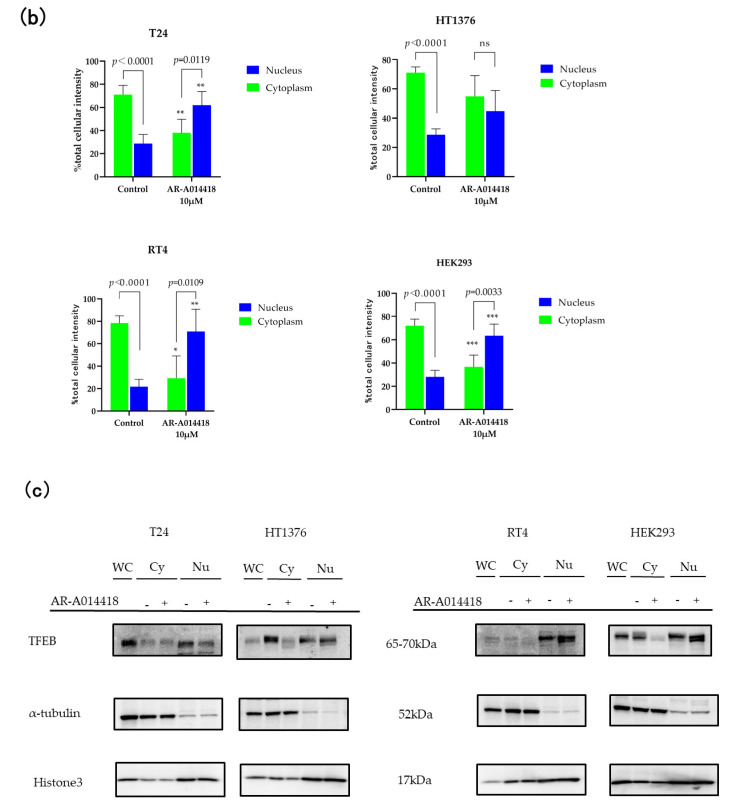
AR-A014418 results in the nuclear translocation of TFEB. (**a**) Immunofluorescence analysis was performed to study the co-localization of TFEB relative to Hoechst 33342-stained nucleus. Scale bar = 50 µm. (**b**) Quantitative analysis of TFEB intensity in (**a**) using ImageJ. Columns, mean; bars, SD. *p* values are indicated above the pairwise brackets. * *p* < 0.05, ** *p* < 0.01, *** *p* < 0.001, ns—not significant; compared to control cells. Statistical analysis was performed using Student’s *t*-test. (**c**) Cytoplasmic and nuclear fractions were analyzed using Western blot analysis to study the levels of TFEB, α-Tubulin (cytoplasmic control), and Histone3 (nuclear control). The original blots are presented in Supplementary Figure S4i–l.

We analyzed the transcriptional regulatory network of the TFEB nucleus translocation upon cell treatment with AR-A014418. We constructed cells expressing the GFP-TFEB fusion proteins and assessed the TFEB nuclear translocation via fluorescent microscopy. All four GFP-TFEB expressed cells were incubated in this study with 0 (control) and 10 μM AR-A014418 for 24 h. Based on the fluorescent microscopy analysis, TFEB is more abundant in the cytoplasm relative to the Hoechst 33342-stained nucleus in the control condition. On the other hand, treatment with AR-A014418 resulted in aggregates of granular puncta of TFEB in the nucleus, overlapping with Hoechst staining (Figure 4a). The intracellular localization of the TFEB intensity was demonstrated to be significantly (*p* < 0.0001) higher in the cytoplasm relative to the nucleus under the control conditions. On the other hand, after treatment with AR-A014418, there was an increase in the nuclear TFEB intensity and a decrease in the cytosolic TFEB intensity (Figure 4b). The nucleus translocation was further confirmed via nucleus cytosol separation assay and detected through the Western blot analysis in Figure 4c.

The data in Figure 4 indicate that the treatment of BC cells with AR-A014418 leads to the nuclear translocation of TFEB, the master transcriptional regulator of autophagy.

### 3.5. The Role of the AMP-AMPK-ULK1 Pathway in GSK-3 Inhibition-Induced Autophagy

**Figure 5:**
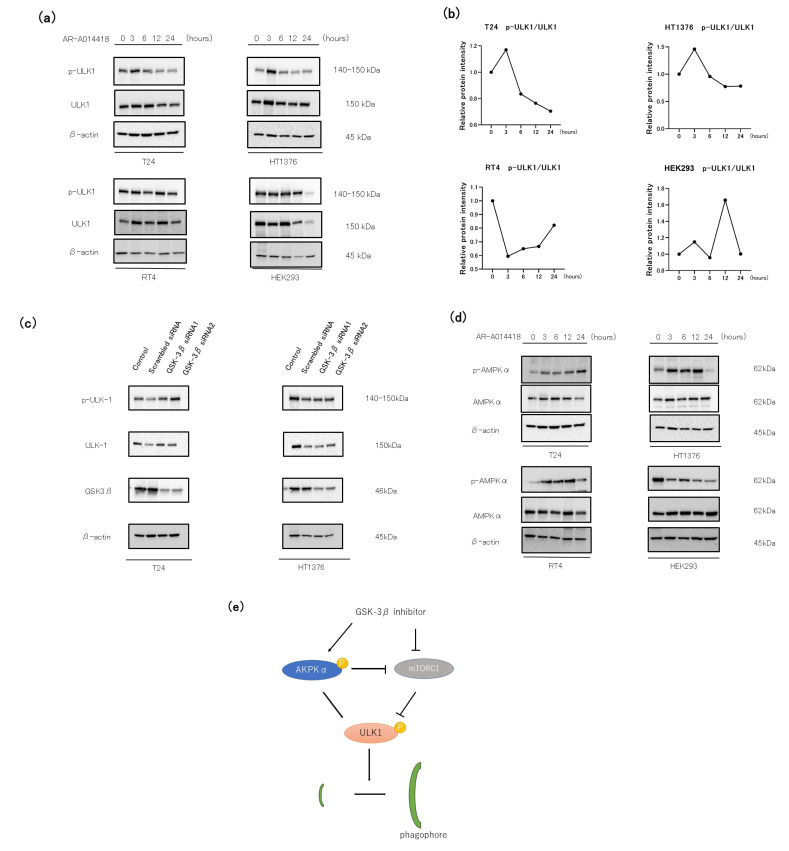
AMPK-ULK1 signaling pathway through the AR-A014418 induces autophagy. Bladder cancer cells were treated with 10 µM AR-A014418 at the indicated time (0, 3, 6, 12, and 24 h). (**a**) Western blot analysis of phosphorylated ULK1 (p-ULK1), total ULK1 (ULK1), and β-actin as a loading control. The original blots are presented in Supplementary Figure S4m–p. (**b**) Relative protein intensity was quantified using ImageJ. (**c**) Western blot analysis of p-ULK1 and ULK1 in siGSK-3β-transfected bladder cancer cells. The original blots are presented in Supplementary Figure S4q,r. (**d**) The expression of phosphorylated AMPKα (p-AMPKα) and total AMPKα (AMPKα) proteins were determined using Western blot analysis. The original blots are presented in Supplementary Figure S4s–v. AMPK-ULK1 signaling pathway through the AR-A014418 induces autophagy. (**e**) Schematic illustration of the induction of autophagy by GSK-3β inhibitor through the AMPK-ULK1 signaling pathway.

We investigated the effects of AR-A014418 on ULK1 and AMPK, the signal-sensing kinases involved in autophagy initiation. Autophagy is elicited in cells through the induction of ULK1 via the phosphorylation of AMPK or the inhibited mTOR [17,34]. The initial stage of phagophore formation is the most complex step in the process of autophagic flux, in which various functional units are involved, including the ULK1 complex. In the present study, elevated p-ULK1 levels are induced with a peak at 3 h of treatment in T24 and HT1376. However, we observed no consistent trend in RT4 and HEK293 (Figure 5a,b), a low grade UC and normal cells, respectively. The GSK3β-specific siRNAs increased the level of p-ULK1 in T24, and the p-ULK1 levels did not change in HT1376 compared to the negative control (Figure 5c).

Then, we also investigated the phosphorylation of AMPK. Elevated p-AMPKα levels are induced with a peak at 24 h of treatment in T24, and a peak at 12 h of treatment in HT1376 and RT4. Unlike the BC cells, HEK293 had the highest expression level of p-AMPKα in the control, and it decreased after 12 h of AR-A014418 treatment (Figure 5d). These data are in consensus with previous reports showing AMPK activation and autophagy after treatment with structurally different GSK-3 inhibitors [35,36].

## 4. Discussion

Autophagy is a promising target for cancer therapy, and the induction of autophagy-associated cytotoxic death by blocking autophagy flux has been recognized as a novel cancer therapeutic strategy [37,38,39]. Previously, we demonstrated that GSK-3β inhibition had an antitumor effect, and treatment with GSK-3β inhibitors resulted in autophagy in BC cells in our laboratory [10].

The therapeutic resistance process in the drug treatment of BC remains to be elucidated, and studies examining GSK-3β inhibitors from the standpoint of autophagy mechanisms have not yet been reported in BC. Therefore, we focused on autophagy, which is thought to contribute to the resistance to existing therapies, and examined whether GSK-3β inhibitors, in combination with autophagy inhibitors, could be a new therapeutic approach for treating BC.

Marchand B. et al. demonstrated that GSK-3 inhibition (CHIR99021 or SB216763) induces pro-survival signals through the increased activity of the autophagy/lysosomal network in pancreatic cancer cells [40]. Russi S. et al. demonstrated that GSK-3 inhibitor (CHIR99021) induces cell cycle arrest, mitotic catastrophe, and autophagic response, resulting in reduced cell proliferation in ES cells [41].

Similarly, we also found that GSK-3β inhibitors (AR-A014418 and TDZD-8) suppress cell proliferation in T24, HT1376, RT4, and HEK293 as observed by the MTS assay (Figure 1 and Supplementary Figure S1). Furthermore, we observed the decreased expression of p62 and increased expression of LC3B and Beclin-1 via treatment with GSK-3β inhibitors. The genetic depletion of GSK-3β by siRNA also lead to similar results, although the results were less pronounced than for pharmacological inhibition (Figure 2b and Figure 3a).

Autophagy has two opposite functions in cancer therapy, a process known as protective autophagy [11,27,42], and type II programmed cell death, which is referred to as autophagic cell death [43,44,45,46,47]. Therefore, it is essential to identify how autophagy behaves in each cancer treatment. Many studies over the past decade have shown that autophagy in BC is inhibited by autophagy inhibitors, including chloroquine, 3MA, and RNA interference [12,42]. Schlutermann et al. treated BC with cisplatin and chloroquine and demonstrated that autophagy enhances cisplatin cytotoxicity [11]. Wang et al. also reported that radiotherapy activates autophagy in BC, and that subsequent cytoprotective autophagy is strongly associated with radiation resistance, further suggesting that the inhibition of autophagy by chloroquine contributes to enhanced radiosensitivity [27].

The combination of chloroquine or its derivatives and chemotherapeutics has been used in phase I and II clinical trials for a variety of tumors [48,49]. Additionally, previous reports showed that GSK-3β inhibitors potentiated the antitumor effect of gemcitabine and cisplatin; in addition, chloroquine potentiated the antitumor effect of GSK-3β inhibitors in BC cells [10]. Our results of the MTS assay show that GSK-3β inhibition-induced autophagy can enhance lethality with chloroquine in BC. These results suggest that GSK-3β inhibition-induced autophagy acts as a pro-survival signal in BC.

TFEB can be phosphorylated by GSK-3 at residues S134 and S138, leading to cytoplasmic retention, whereas GSK-3 inhibition leads to TFEB nuclear translocation [50]. GSK-3 inhibition leads to TFEB dephosphorylation, which correlates with TFEB dislocation from 14-3-3 chaperones and TFEB nuclear localization in the pancreatic cancer cell [40]. Our study provides partial support for the role of GSK-3β in the regulation of TFEB in the BC cell. Our data showed that TFEB-GFP in T24 translocated from the cytoplasm to the nucleus after GSK-3β inhibitor treatment, but the Western blot analysis showed that the cytoplasmic TFEB protein was unchanged after treatment, and the nuclear translocation was reversed with a decrease in the TFEB protein (Figure 4c). One possible explanation for this result is the hypothesis that T24 is a high grade BC, and therefore retains TFEB in the nucleus from a steady state, but further experiments are needed to prove this.

The AMPK pathway is an important upstream signal, as a key cellular energy sensor, of autophagy activation [51]. When the cellular AMP/ATP ratio increases, AMPK is activated and mTORC1 is suppressed [52]. mTORC1 lies upstream of the ULK1 complex and negatively regulates this complex. AMPK activation and mTORC1 suppression lead to the phosphorylation of ULK1, resulting in phagophore and autophagosome formation [53]. In addition, previous studies reported that treating cells with GSK-3 inhibitors inhibited mTORC1 activity and increased autophagic flux [54] (Figure 5e).

Our data showed that the p-ULK1/ULK1 protein levels increased as early as 3 h after GSK-3β inhibitor (AR-A014418) treatment in the high and intermediate grade BC cells and decreased with time, whereas no such trend was observed in the low grade BC cells or normal kidney cells. In the present study, from an autophagy perspective, treatment with GSK-3β inhibitors potentiate apoptosis and proliferation in the BC cell. However, the in vitro culture cannot completely simulate the in vivo internal environment. Therefore, in vivo experiments will be required to be conducted in a future study. In addition, bioinformatic analysis such as BCG and IL-2 models for bladder cancer treatment [55] could also be useful. The results of our study and observation are in concordance with similar studies [56,57,58], and this approach presents a rationale to overcome drug resistance from an autophagy perspective.

Finally, phase I and II clinical trials of GSK-3β inhibitor are being conducted for pancreatic cancer and gliomas. If this is clinically applied in the future, it is expected to contribute significantly to BC treatment.

Our results suggest that autophagy inhibition can potentiate the effect of GSK-3β inhibition by abrogating pro-survival autophagy activation. Thus, combination therapy with these two small molecules can be advantageous in BC patients.

## 5. Conclusions

We found that GSK-3β inhibitor induces pro-survival autophagy, and targeting autophagy potentiates GSK-3β inhibition-induced apoptosis and retarded proliferation in BC cells. This indicates that GSK-3β inhibitor can be used as an autophagy-targeted drug, and autophagy blockade has the potential to be an effective interventional strategy for addressing cancer progression and overcoming therapeutic resistance in addition to existing treatment. Despite there being only a few clinical trials targeting autophagy in BC or using GSK-3β inhibitors, they are expected to make a significant contribution to BC treatment if clinically applied in the future.

## Data Availability

All data generated or analyzed during this study are included in this published article and its Supplementary Materials.

## Supplementary Materials

The following supporting information can be downloaded at https://www.mdpi.com/article/10.3390/curroncol30060406/s1, Supplementary Figure S1: Relative cell viability of bladder cancer cells and human embryonic kidney 293 (HEK293) cells treated with TDZD-8 for 72 h; Supplementary Figure S2: Effect of TDZD-8 on the expression of autophagy-related proteins; Supplementary Figure S3: Morphological changes in the cells after GSK-3β knockdown using siRNA; Supplementary Figure S4: Full-length images of Western blot analyses and densitometry readings/intensity ratio of each band.
