# Supplementary material for: Targeting Pro-Survival Autophagy Enhanced GSK-3β Inhibition-Induced Apoptosis and Retarded Proliferation in Bladder Cancer Cells
Source: Curr Oncol. 2023 May 28;30(6):5350–65. doi: 10.3390/curroncol30060406 (PMC10297715; doi:10.3390/curroncol30060406)
Supplement: Supplementary file 1 [file curroncol-30-00406-s001.zip › Supplementary Figure S1-S3.pdf]

Targeting Pro-Survival Autophagy Enhanced GSK-3β Inhibition-Induced Apoptosis and Retarded Proliferation in Bladder Cancer Cells

Supplementary Materials

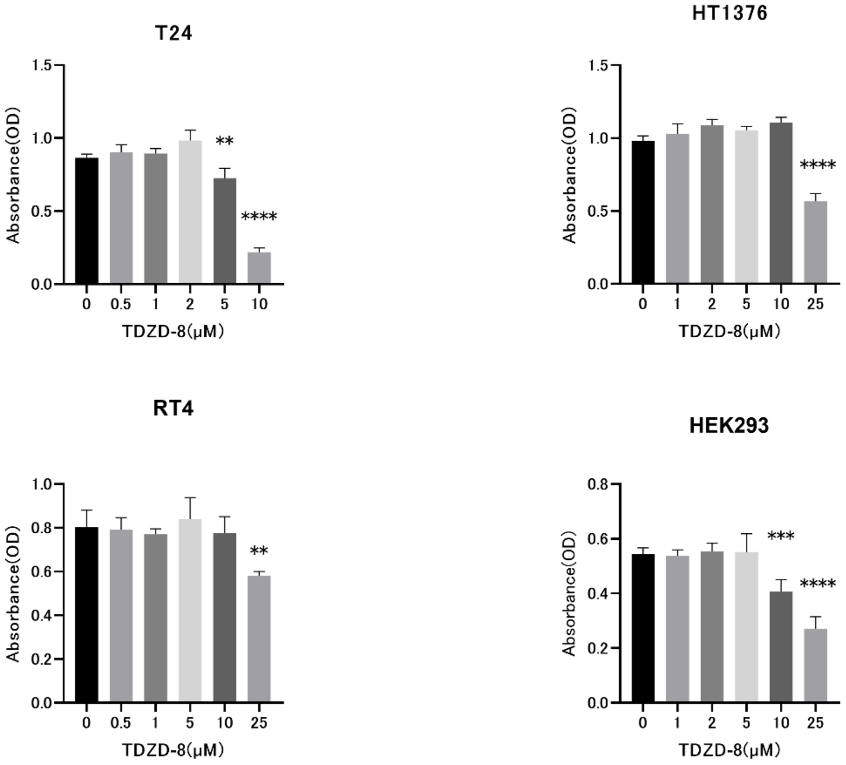

Supplementary Figure S1. Bladder cancer cells and human embryonic kidney 293(HEK293) cells were treated with the indicated concentration of TDZD-8 for 72 hours. Relative cell viability was measured by MTS assay. OD: optical density. **p<0.01, ***p<0.001, ****p<0.0001; compared to control cells.

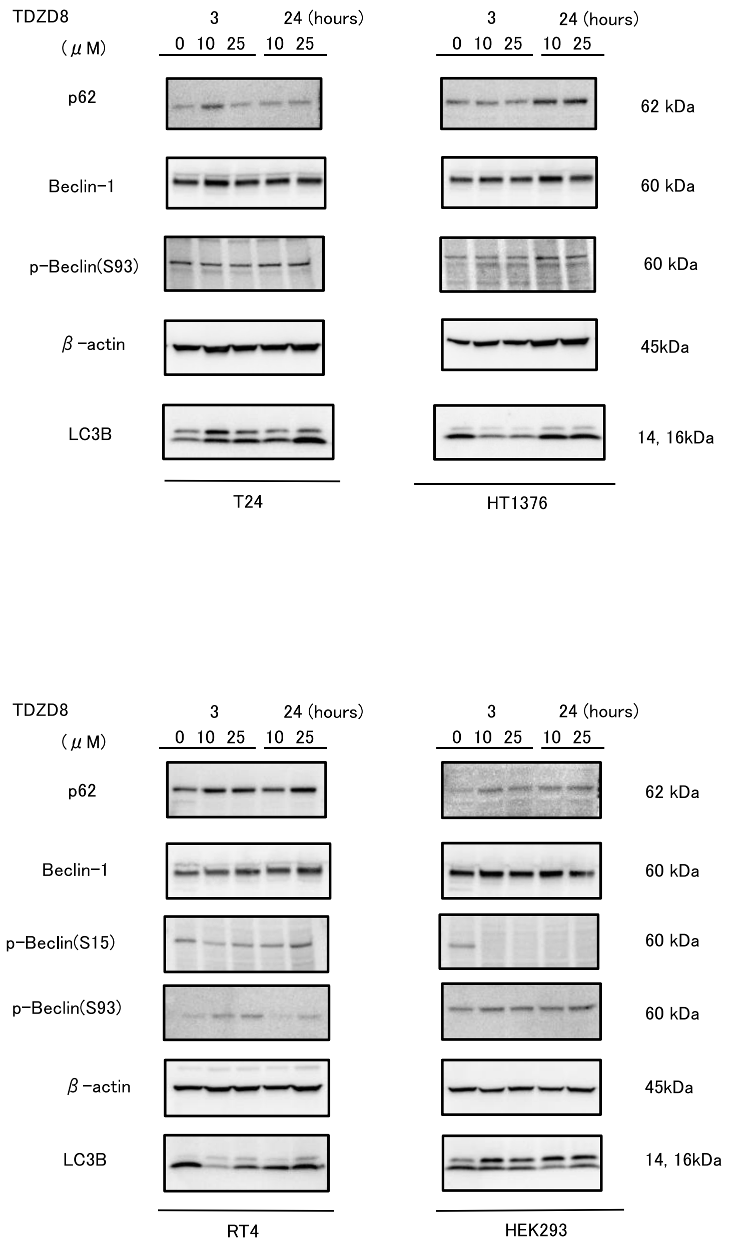

Supplementary Figure S2. Effect of TDZD-8 on the expression of autophagy-related proteins in bladder cancer cells. Western blot analysis of p62, Beclin-1, p-Beclin(s15, s93) and LC3B. β-actin was used as a loading control.

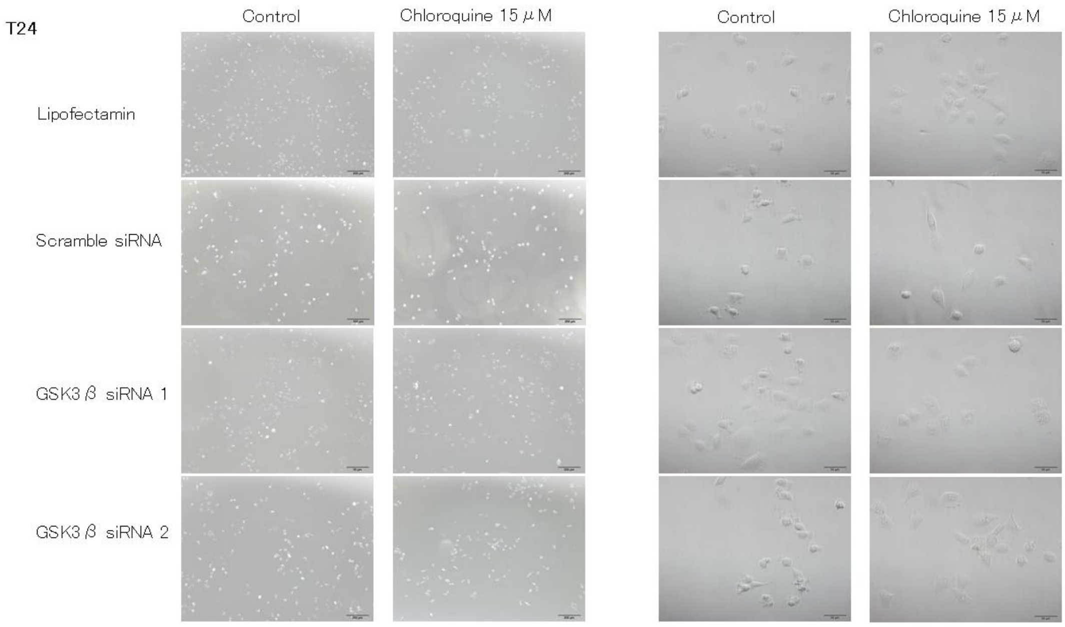
T24
Control
Chloroquine 15 μM
Control
Chloroquine 15 μM
Lipofectamin
Scramble siRNA
GSK3β siRNA 1
GSK3β siRNA 2

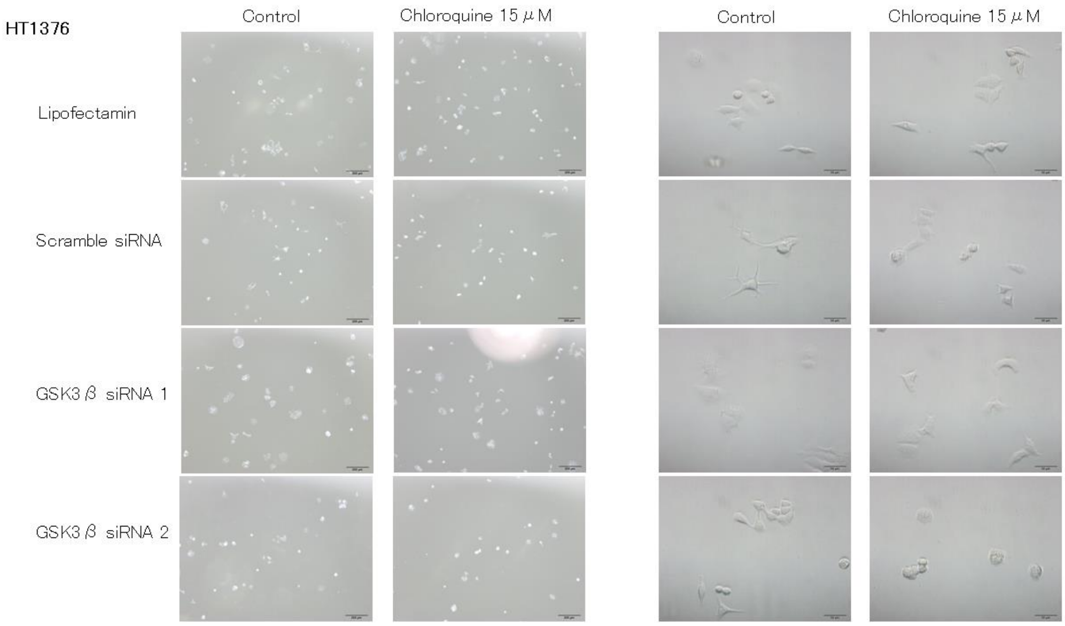
HT1376
Control
Chloroquine 15 μM
Control
Chloroquine 15 μM
Lipofectamin
Scramble siRNA
GSK3β siRNA 1
GSK3β siRNA 2

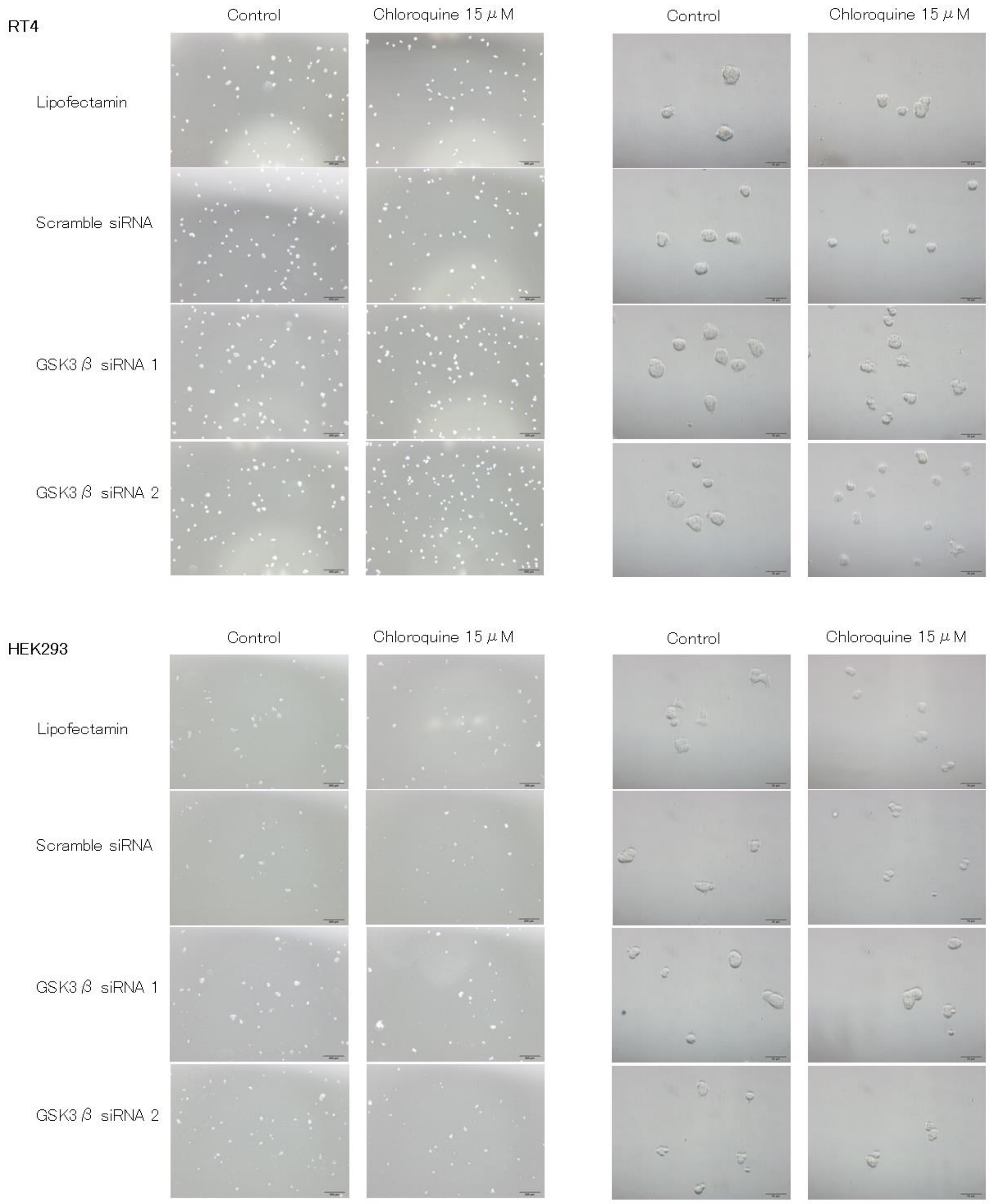

Supplementary Figure S3. Morphological changes in the cells after GSK-3β knockdown using siRNA. siGSK-3β-transfected bladder cancer cells treated with chloroquine for 24 hours showed extensive vacuolation. Scale bar=200 μm(left 2 columns) and 50 μm(right 2 columns).
