# Supplementary material for: Targeting Pro-Survival Autophagy Enhanced GSK-3β Inhibition-Induced Apoptosis and Retarded Proliferation in Bladder Cancer Cells
Source: Curr Oncol. 2023 May 28;30(6):5350–65. doi: 10.3390/curroncol30060406 (PMC10297715; doi:10.3390/curroncol30060406)
Supplement: Supplementary file 1 [file curroncol-30-00406-s001.zip › Supplementary Figure S4.pdf]

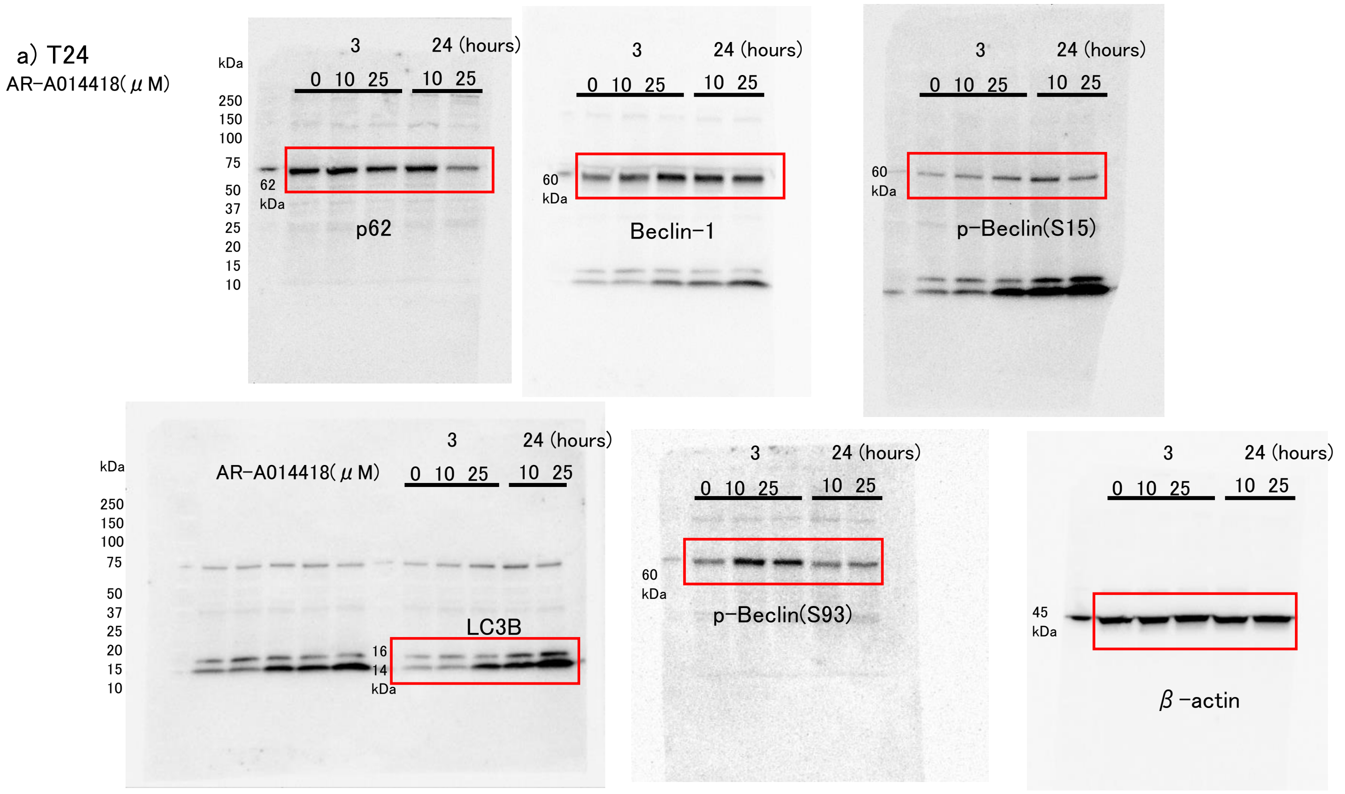

	3		24 (hours)		
	0	10	25	10	25 (µM)
p62/β-actin	0.956	0.903	0.745	1.003	0.331
Beclin-1/β-actin	0.456	0.575	0.837	1.03	0.761
p-Beclin(S15)/β-actin	0.203	0.309	0.428	0.655	0.335
p-Beclin(S93)/β-actin	0.455	0.933	0.759	0.389	0.221
LC3B/β-actin	0.35	0.449	0.894	1.5	1.415

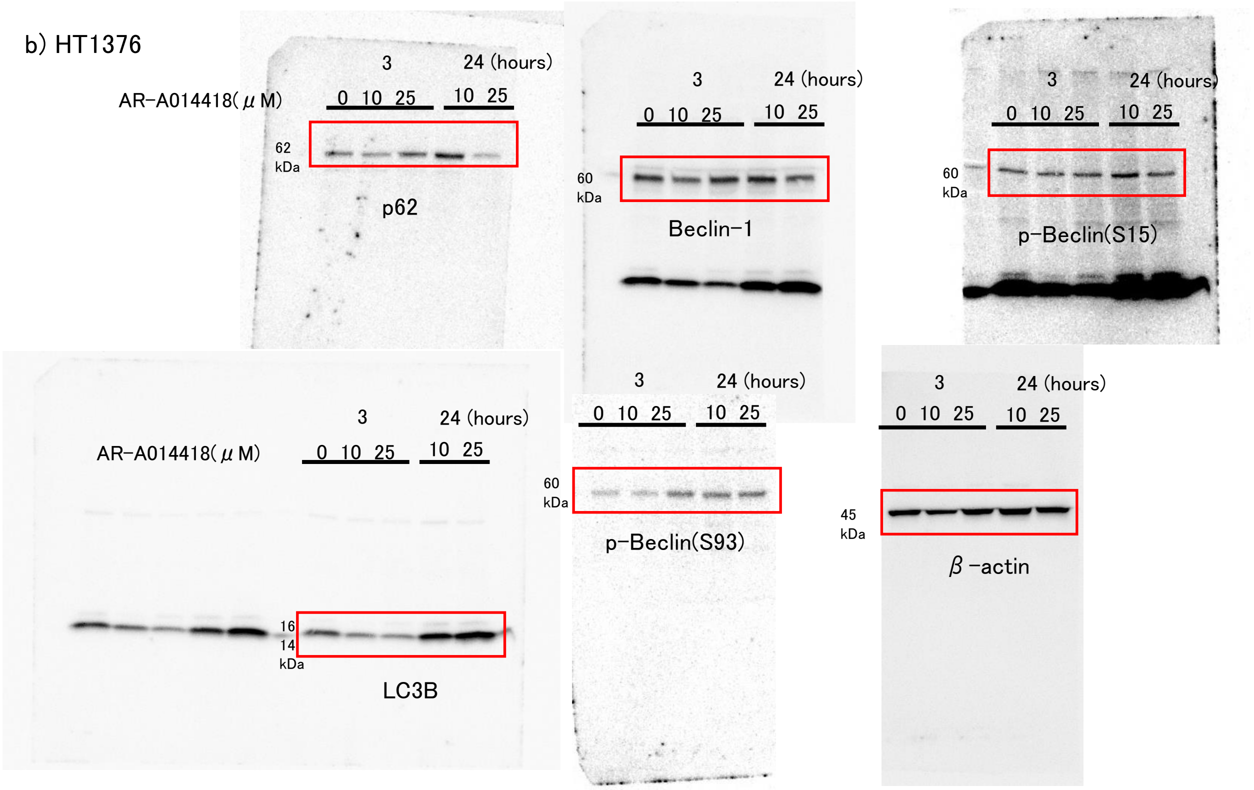

	3		24 (hours)		
	0	10	25	10	25 (µM)
p62/β-actin	0.399	0.346	0.528	0.747	0.174
Beclin-1/β-actin	0.789	0.881	0.850	0.856	0.565
p-Beclin(S15)/β-actin	0.566	0.600	0.465	0.530	0.365
p-Beclin(S93)/β-actin	0.460	0.500	0.881	0.930	0.842
LC3B/β-actin	0.593	0.490	0.388	0.863	1.203

c) RT4

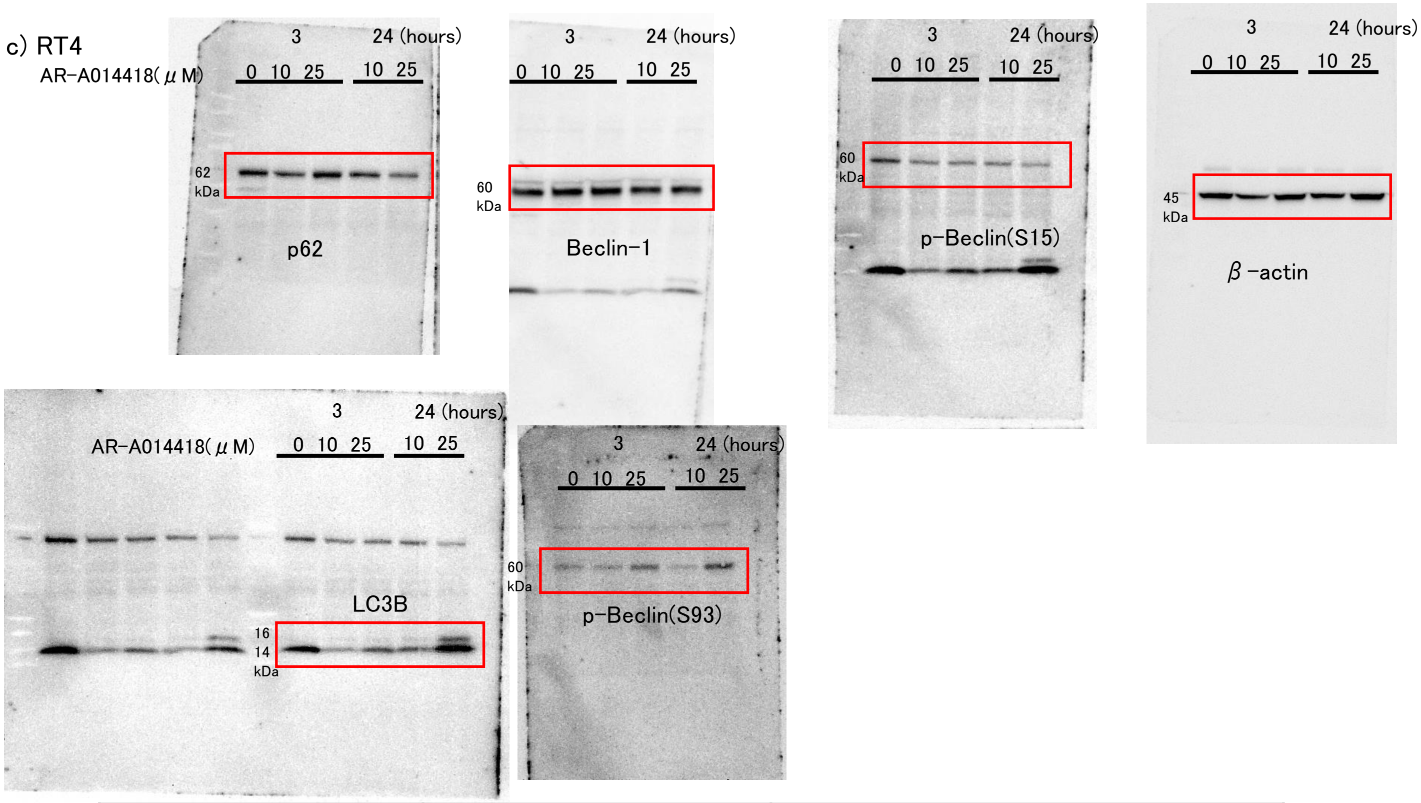

	3		24 (hours)		
	0	10	25	10	25 (μM)
p62/β-actin	0.984	0.882	0.966	0.853	0.421
Beclin-1/β-actin	0.813	1.516	1.434	1.320	0.919
p-Beclin(S15)/β-actin	0.719	0.551	0.420	0.446	0.224
p-Beclin(S93)/β-actin	0.401	0.401	0.632	0.311	0.825
LC3B/β-actin	1.041	0.546	0.618	0.721	1.315

d) HEK293

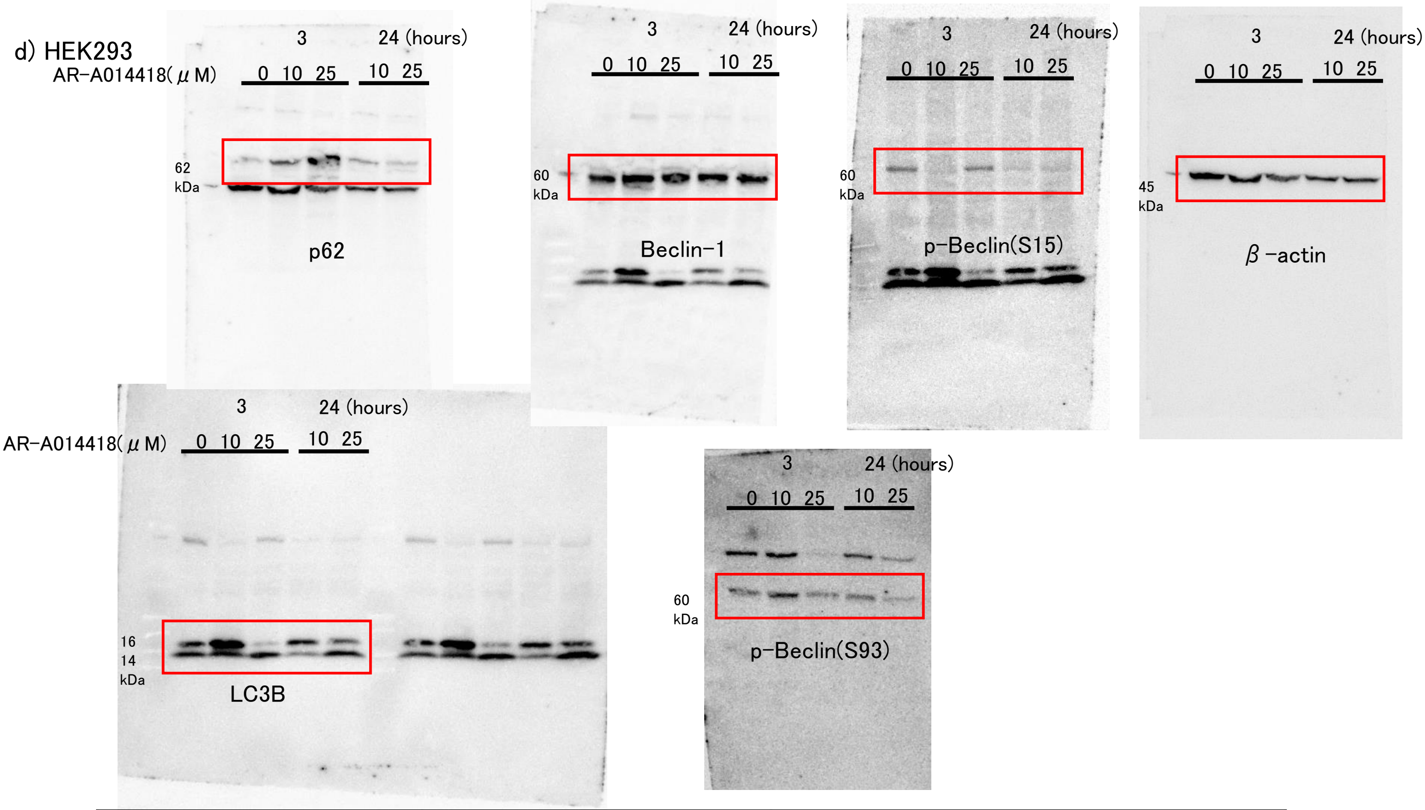

	3		24 (hours)		
	0	10	25	10	25 (μM)
p62/β-actin	0.190	0.619	1.785	0.425	0.223
Beclin-1/β-actin	0.872	1.352	1.613	1.632	1.567
p-Beclin(S15)/β-actin	0.627	0.135	0.813	0.130	0.270
p-Beclin(S93)/β-actin	0.404	0.837	0.680	0.828	0.630
LC3B/β-actin	1.160	2.324	1.509	1.595	1.920

e) T24

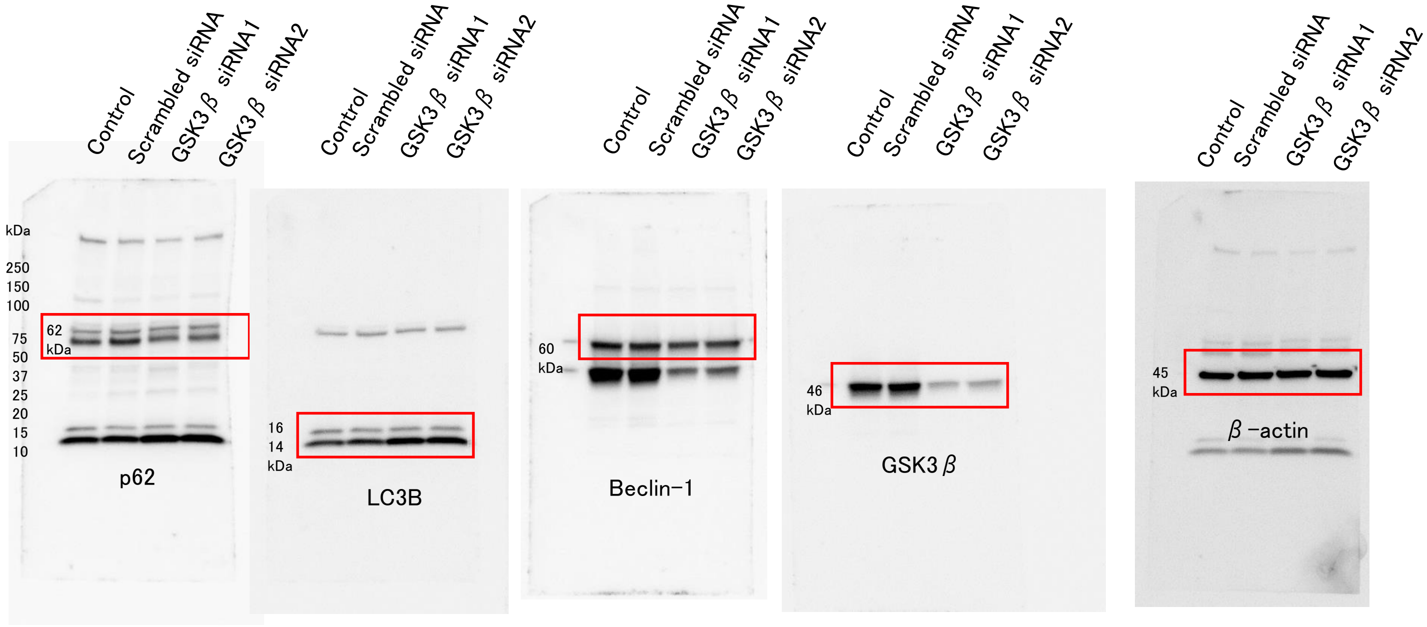

	Control	Scrambled siRNA	GSK-3β siRNA1	GSK-3β siRNA2
p62/β-actin	0.934	1.153	0.776	0.827
Beclin-1/β-actin	0.806	0.635	0.540	0.515
GSK3β/β-actin	1.270	1.204	0.273	0.273
LC3B/β-actin	0.612	0.674	0.932	0.836

f) HT1376

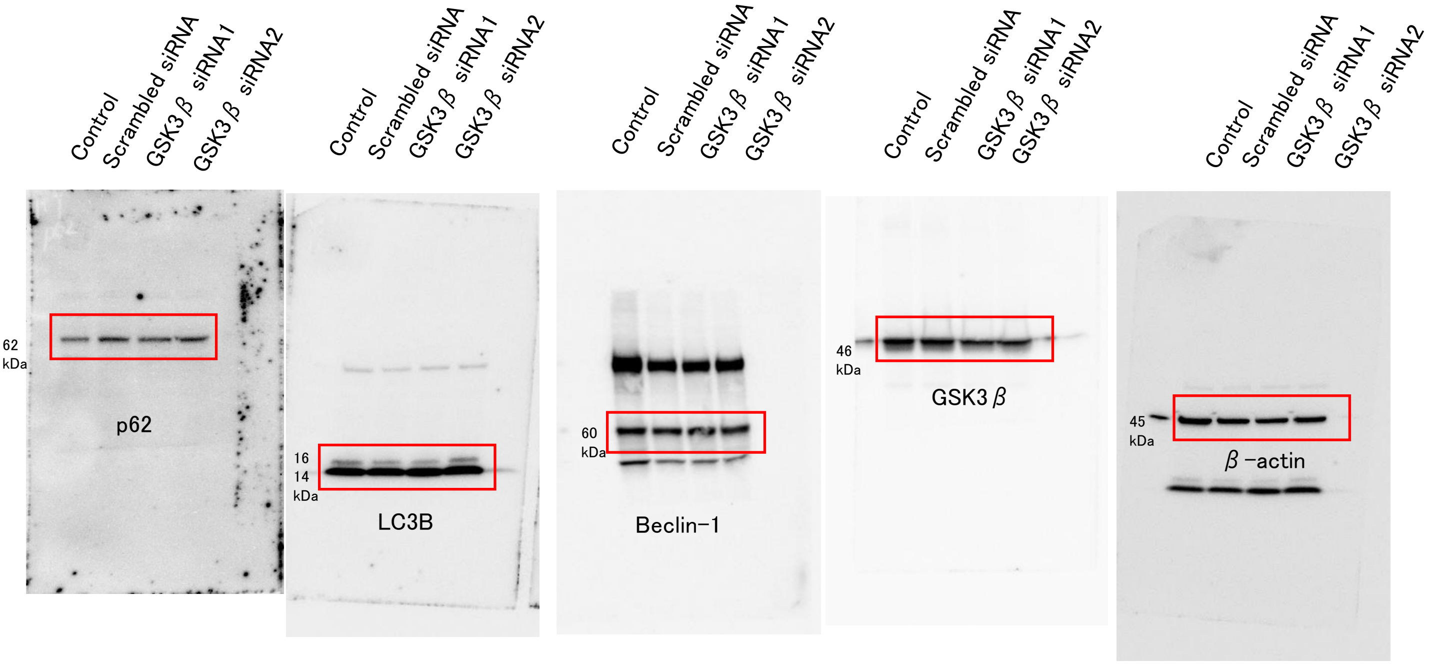

	Control	Scrambled siRNA	GSK-3β siRNA1	GSK-3β siRNA2
p62/β-actin	0.420	0.763	0.735	0.818
Beclin-1/β-actin	1.060	1.134	1.098	1.266
GSK3β/β-actin	1.156	1.331	1.330	1.057
LC3B/β-actin	1.004	1.350	1.675	1.738

g) RT4

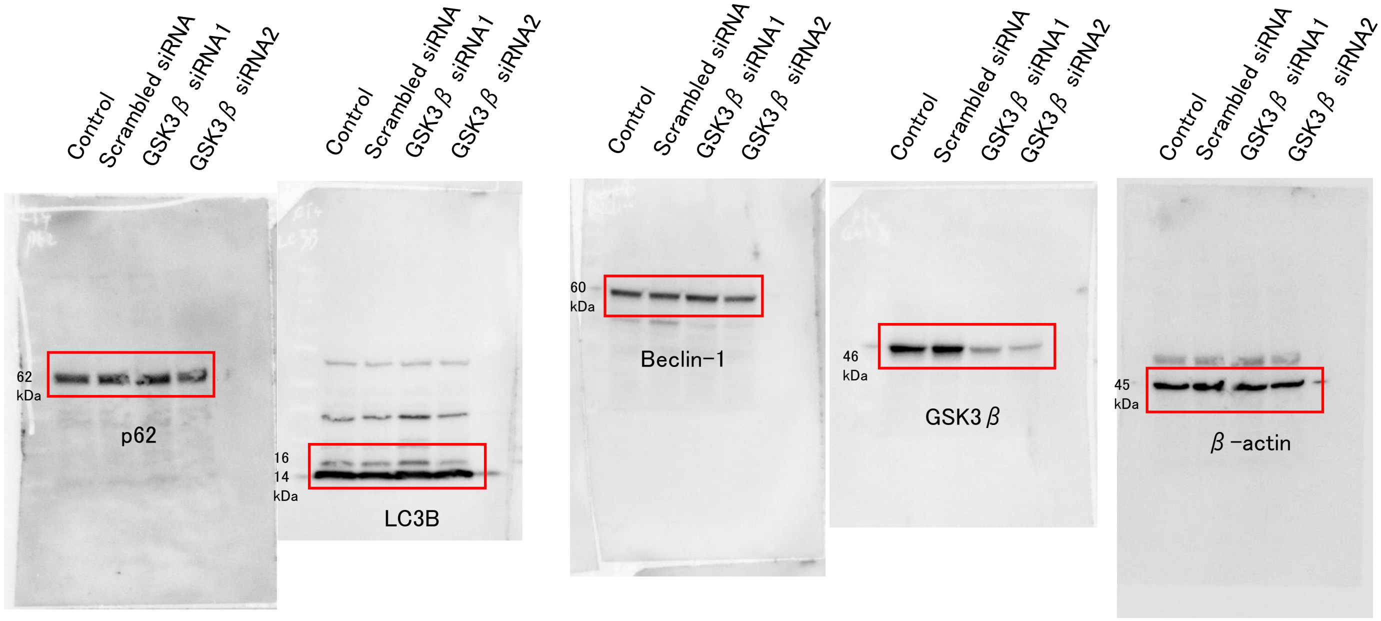

	Control	Scrambled siRNA	GSK-3β siRNA1	GSK-3β siRNA2
p62/β-actin	1.254	1.117	1.368	1.312
Beclin-1/β-actin	1.006	0.920	1.124	0.909
GSK3β/β-actin	1.122	0.965	0.345	0.255
LC3B/β-actin	1.468	1.175	1.494	1.775

h) HEK293

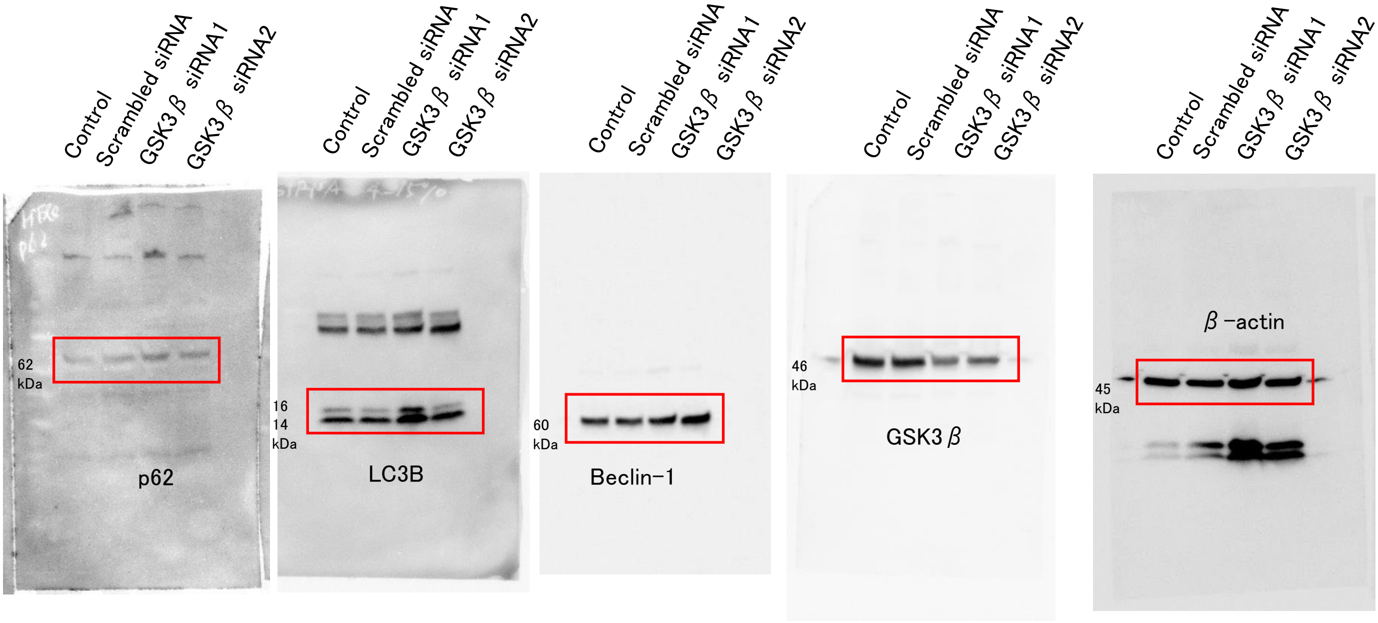

	Control	Scrambled siRNA	GSK-3β siRNA1	GSK-3β siRNA2
p62/β-actin	0.245	0.507	0.542	0.411
Beclin-1/β-actin	0.738	0.755	0.821	1.246
GSK3β/β-actin	1.058	1.162	0.574	0.606
LC3B/β-actin	0.856	0.868	1.268	0.671

i) T24

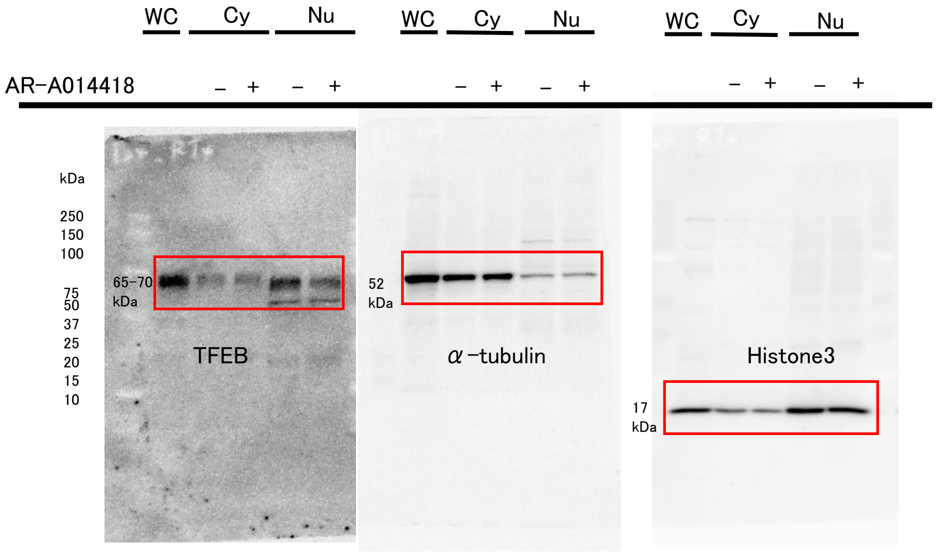

	WC	Cy		Nu	
		ARA-	ARA+	ARA-	ARA+
TFEB/α-tubulin	1.163	0.424	0.477		
TFEB/Histone3				0.960	0.513

j) HT1376

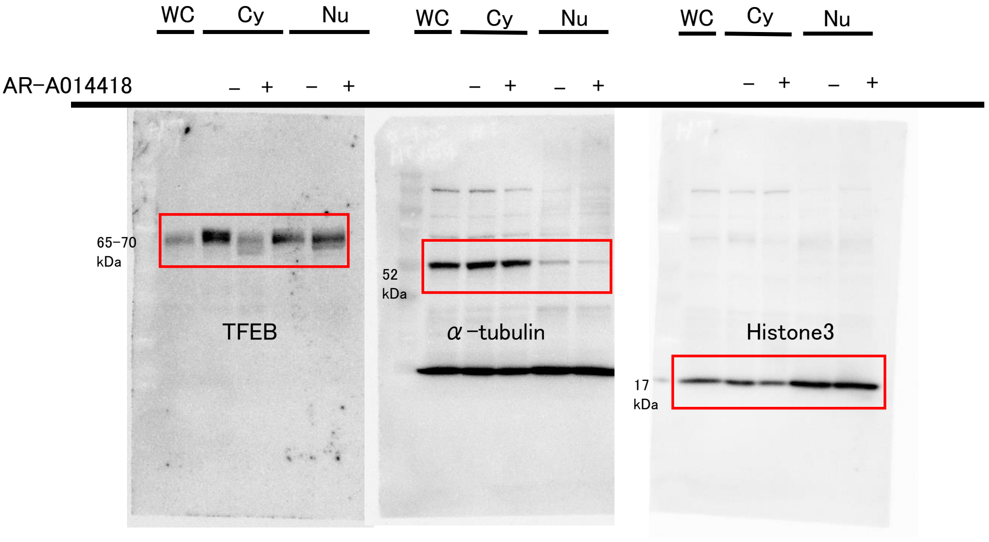

	WC	Cy		Nu	
		ARA-	ARA+	ARA-	ARA+
TFEB/α-tubulin	0.818	2.092	1.030		
TFEB/Histone3				1.445	1.628

k) RT4

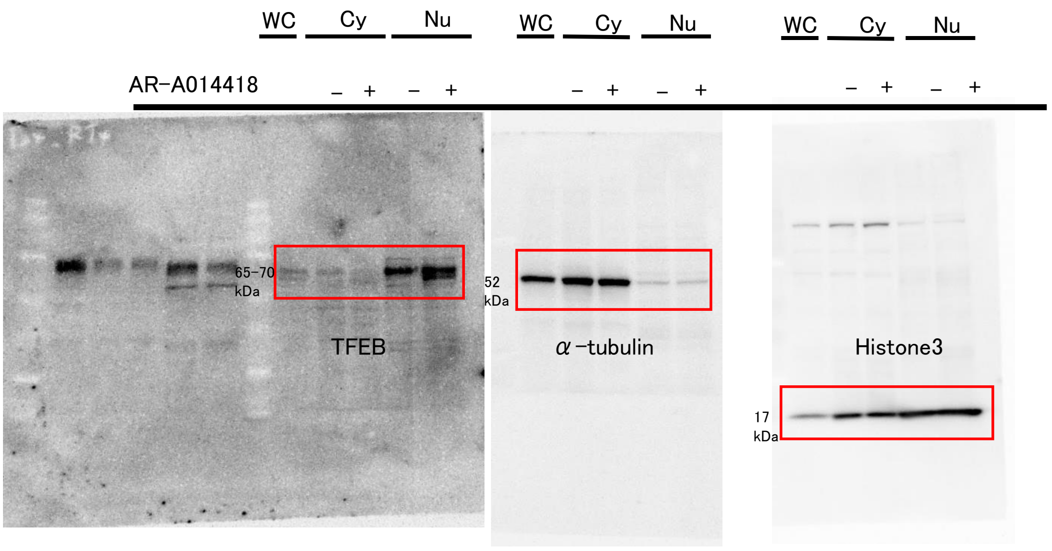

	WC	Cy		Nu	
		ARA-	ARA+	ARA-	ARA+
TFEB/α-tubulin	0.500	0.101	0.138		
TFEB/Histone3				0.879	1.040

l) HEK293

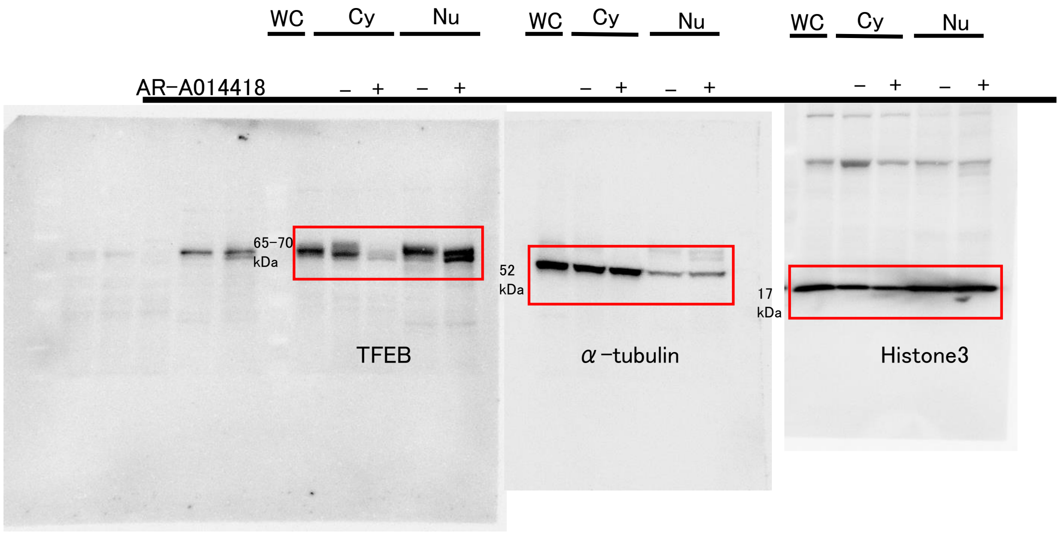

	WC	Cy		Nu	
		ARA-	ARA+	ARA-	ARA+
TFEB/α-tubulin	0.838	0.757	0.310		
TFEB/Histone3				0.967	1.100

m) T24

n) HT1376

AR-A014418 10 μM

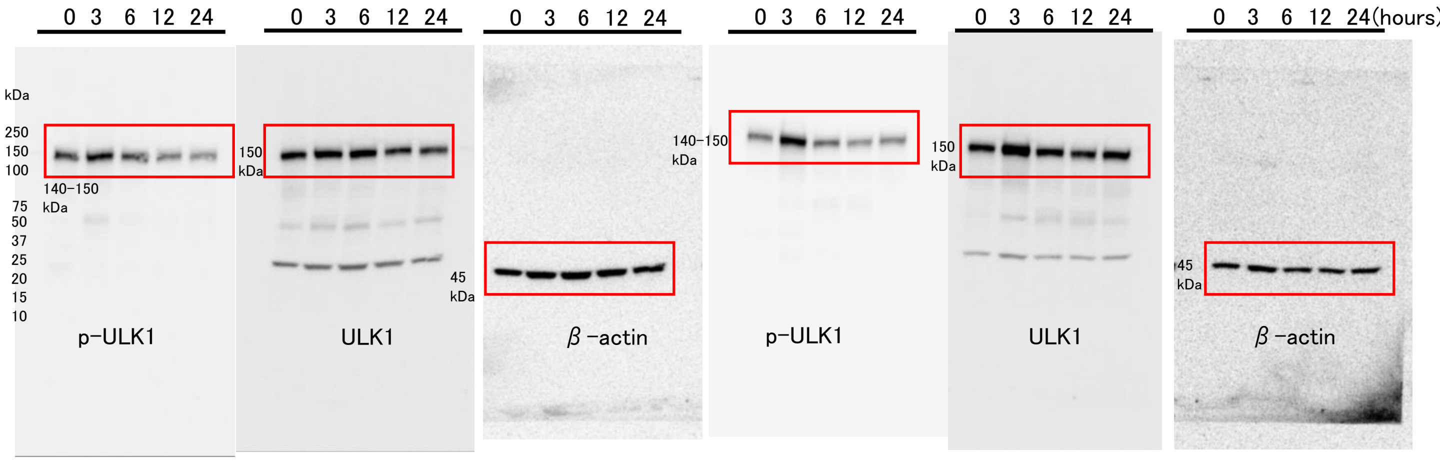

	0	3	6	12	24	0	3	6	12	24(hours)
p-ULK1/ULK1	0.794	0.929	0.662	0.607	0.559	0.580	0.846	0.555	0.449	0.454
p-ULK1/β-actin	0.950	1.115	0.768	0.536	0.499	0.812	1.425	0.908	0.703	0.732
ULK1/β-actin	1.197	1.200	1.160	0.882	0.893	1.400	1.684	1.635	1.567	1.612

o) RT4

p) HEK293

AR-A014418 10 μM

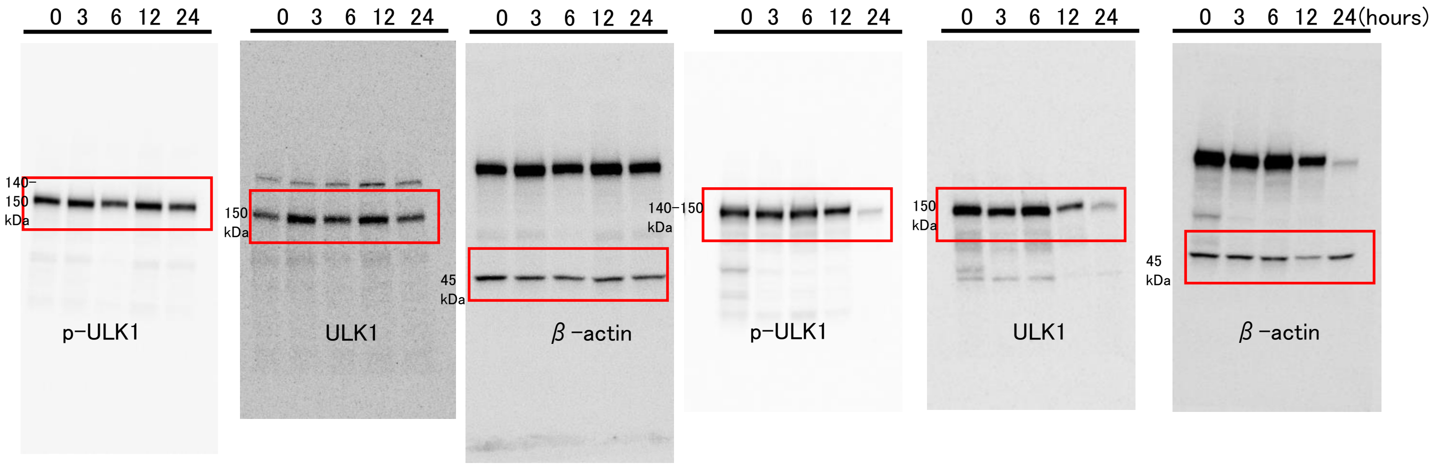

	0	3	6	12	24	0	3	6	12	24(hours)
p-ULK1/ULK1	1.755	1.043	1.139	1.169	1.442	0.940	1.080	0.899	1.559	0.942
p-ULK1/β-actin	1.544	1.887	1.525	1.704	1.608	2.235	2.267	2.719	4.001	0.448
ULK1/β-actin	0.880	1.809	1.339	1.457	1.115	2.378	2.099	3.023	2.567	0.476

q) T24

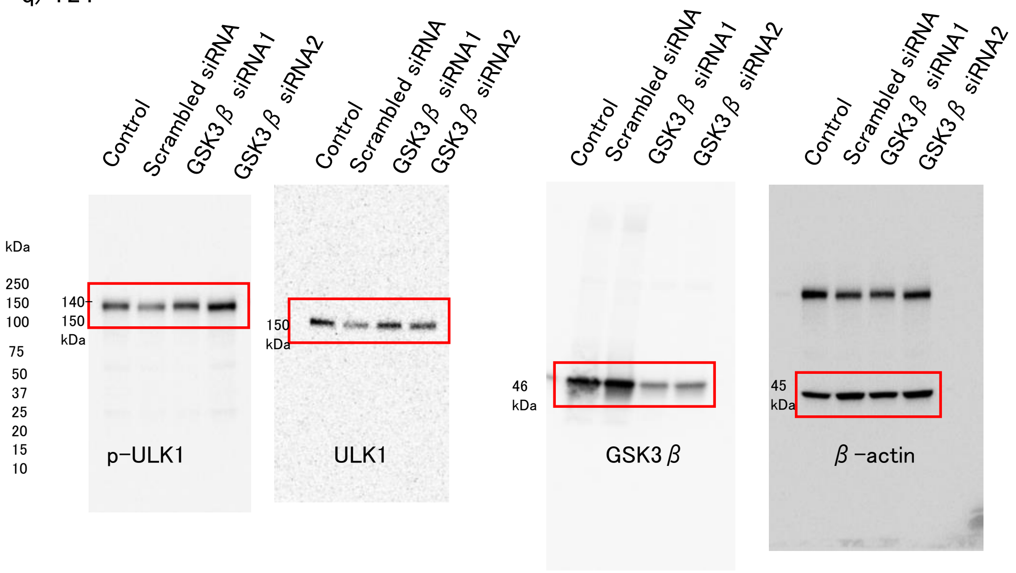

	Control	Scrambled siRNA	GSK-3β siRNA1	GSK-3β siRNA2
p-ULK2/β-actin	0.906	0.512	0.916	1.049
ULK1/β-actin	1.001	0.417	0.882	0.700
GSK3β/β-actin	0.703	0.390	0.227	0.190

r) HT1376

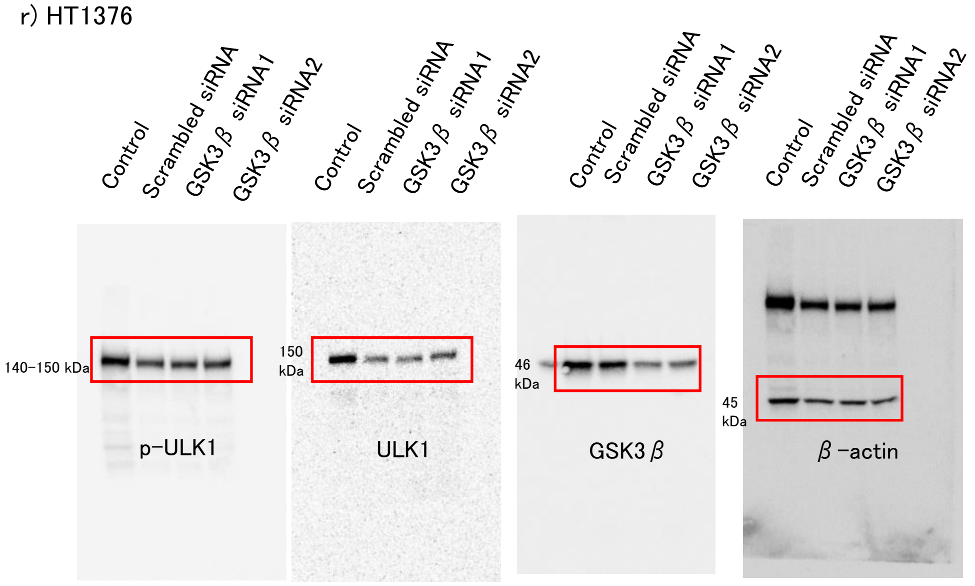

	Control	Scrambled siRNA	GSK-3β siRNA1	GSK-3β siRNA2
p-ULK2/β-actin	1.491	1.381	1.229	1.743
ULK1/β-actin	1.501	0.901	1.020	1.528
GSK3β/β-actin	1.265	1.844	1.055	1.457

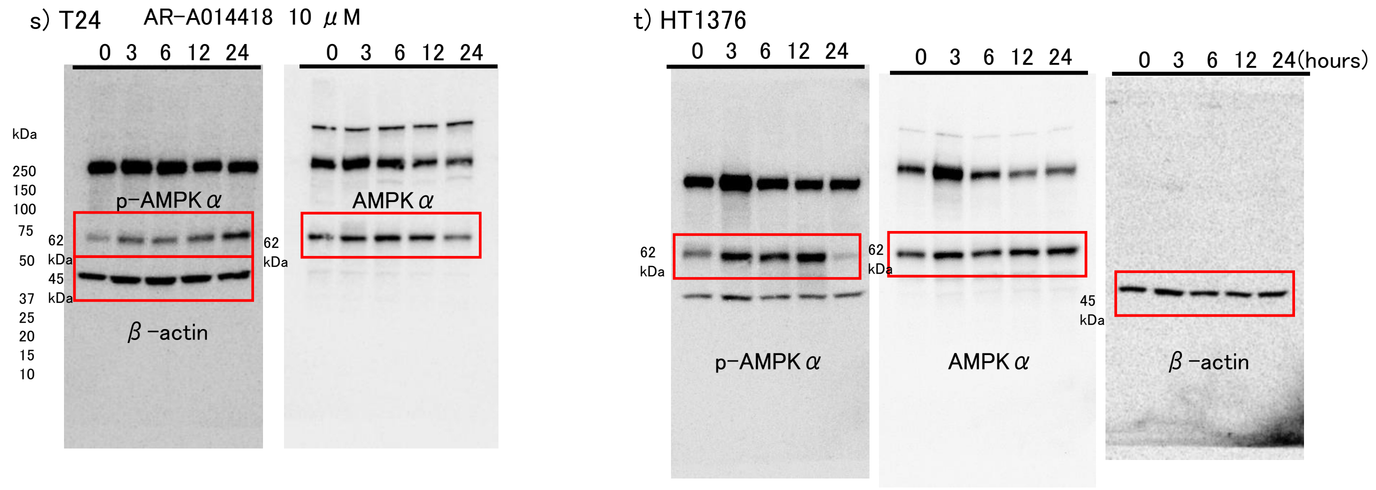

	0	3	6	12	24	0	3	6	12	24(hours)
p-AMPKα/β-actin	0.496	0.676	0.592	0.762	1.049	1.280	1.552	1.718	1.844	0.198
AMPKα/β-actin	0.958	0.724	0.907	0.806	0.598	1.401	1.378	1.426	1.870	1.484

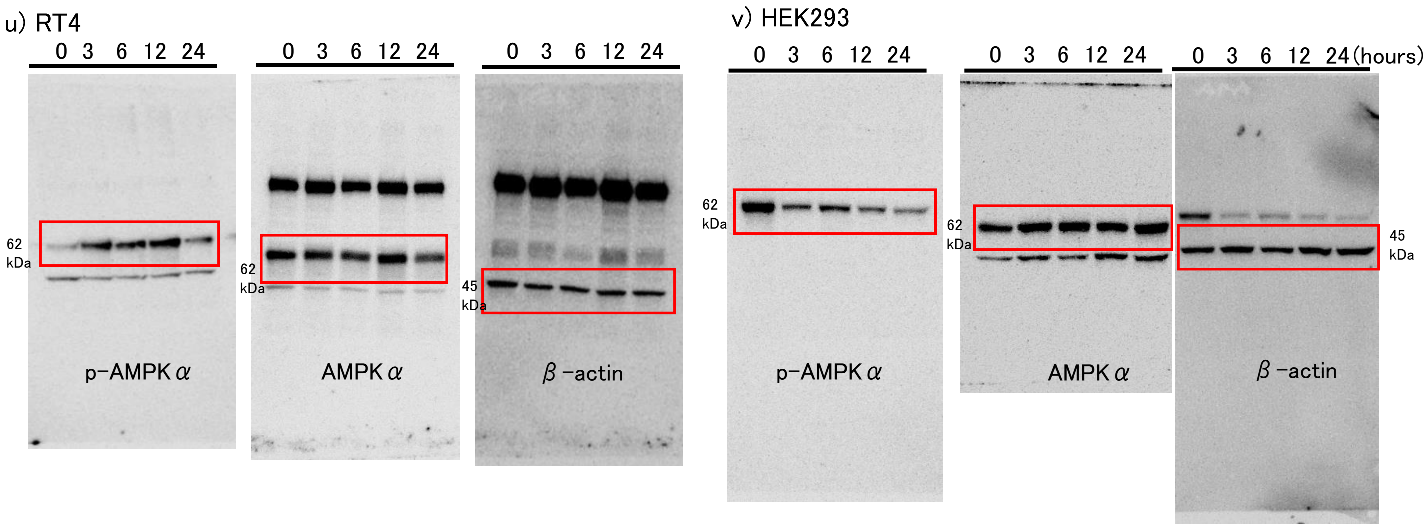

	0	3	6	12	24	0	3	6	12	24(hours)
p-AMPKα/β-actin	0.264	1.185	1.088	1.661	0.969	1.927	0.708	1.109	0.537	0.517
AMPKα/β-actin	1.194	1.309	1.028	1.172	0.858	1.500	1.416	1.779	1.333	1.638

Supplementary Figure S4. Full-length images of western blot analyses and densitometry readings/intensity ratio of each band. a)-d) Full-length images presented in Figure 2b. e)-h) Full-length images presented in Figure 3a. i)-l) Full-length images presented in Figure 4c. m)-p) Full-length images presented in Figure 5a. q), r) Full-length images presented in Figure 5c. s)-v) Full-length images presented in Figure 5d. The red square depicts the area presented in the main figures.
